# Dietary *Klebsormidium* sp. Supplementation Improves Growth Performance, Antioxidant and Anti-Inflammatory Status, Metabolism, and Mid-Intestine Morphology of *Litopenaeus Vannamei*

**DOI:** 10.3389/fnut.2022.857351

**Published:** 2022-05-12

**Authors:** HaoHang Fang, ZhenXiao Zhuang, LuoDong Huang, Wei Zhao, Jin Niu

**Affiliations:** ^1^State Key Laboratory of Biocontrol, Guangdong Provincial Key Laboratory for Aquatic Economic Animals and Southern Marine Science and Engineering Guangdong Laboratory (Zhuhai), School of Life Sciences, Sun Yat-sen University, Guangzhou, China; ^2^Institute of Marine Research, Bergen, Norway; ^3^College of Life Science and Technology, Guangxi University, Nanning, China

**Keywords:** *Klebsormidium* sp., *Litopenaeus vannamei*, growth performance, antioxidant, hepatopancreas health

## Abstract

Filamentous microalga *Klebsormidium* sp. has huge potential to become a natural and healthy additive in aquatic feed since it contains various bioactive nutrients, such as linoleic acid (LA), carotenoids, and chlorophylls. Therefore, an eight-week feeding experiment was performed to evaluate the effects of dietary *Klebsormidium* sp. on the growth performance, antioxidant and anti-inflammatory status, metabolism, and mid-intestine morphology of *Litopenaeus vannamei*. Two isonitrogenous and isolipid diets supplemented with and without 5% *Klebsormidium* sp. were prepared. Results showed that *L. vannamei* fed with *Klebsormidium* sp. had better growth performance and feed utilization by optimizing mid-intestine morphology and improving the carbohydrate metabolism. In addition, *Klebsormidium* sp. also enhanced the antioxidant capacity of *L. vannamei* by downregulating antioxidant parameters (hepatopancreas T-SOD, hepatopancreas GSH-PX, hemolymph T-SOD, hemolymph MDA) and RNA expression levels of antioxidant genes (*gsh-px* and *cat*). Furthermore, the supplementations of dietary *Klebsormidium* sp. significantly improved hepatopancreas health by downregulating RNA expression levels of pro-inflammatory related genes (*relish* and *rho*). Therefore, a dose of 5% *Klebsormidium* sp. is recommended for the daily diet of *L. vannamei* to improve the growth performance, antioxidant and anti-inflammatory status, metabolism, and mid-intestine morphology of shrimp.

## Introduction

High-density farming and environmental pollution are two main reasons that limit the development of the shrimp industry since they lead to the growth of pathogenic microorganisms in water, making shrimp vulnerable to bacterial diseases (1, 2). To address this issue, antibiotics are widely used in aquaculture to prevent and treat bacterial diseases (3). However, the problem of long-term antibiotic exposure in aquatic animals is the antibiotic residue in aquatic products. This causes adverse effects on humans when seafood with numerous antibiotic residues is consumed. For example, it reduces the effectiveness of antibiotics in human if they face infections (4), and changes the diversity of gut microbiota (5). In this situation, there is a need to identify healthy and economic additives for substituting the use of antibiotics in the aquaculture field.

Crustaceans are generally considered to have a low ability to biosynthesize *de novo* polyunsaturated fatty acids (PUFAs). They lack Δ12 and Δ15 desaturase enzymes, which makes it hard for them to convert the oleic acid (OA) into linoleic acid (LA) and linolenic acid (LNA) (6). Therefore, shrimps require essential fatty acids (EFAs) in their daily diet. Besides, the supply of PUFAs at optimal levels and ratios in their diet is also beneficial for their growth performance (7), anti-inflammatory status (8, 9), and resistance to diseases (10–13). Therefore, substances rich in PUFAs might be an excellent choice as aquaculture feeding additives.

*Klebsormidium* sp., a filamentous microalga that is rich in LA with a rapid growth rate (14), can exist in typical extreme environments such as drought (15, 16) and deep freeze climate (17, 18). Except for n-6 PUFAs, *Klebsormidium* sp. is also a rich source of various bioactive pigments (carotenoids and chlorophylls) (19, 20). Therefore, this microalga fits the criterion of healthy and natural additive in aquatic feed. However, until recently, knowledge about the effect of dietary *Klebsormidium* sp. on aquatic animals is unclear.

*Litopenaeus vannamei*, belonging to the genus *Penaeus*, is mainly found in the tropical waters along the Pacific coast of the United States (21). For nearly two decades, *L. vannamei* has gained more attention and is widely cultured in China, India, and some Southeast Asian countries due to its delicious meat and high nutritional value (22). However, with the development of green aquaculture, the use of antibiotics is strictly limited during farming worldwide. Therefore, it is necessary to identify a healthy and cost-effective aquatic additive for substituting antibiotics, which are used for improving the health of shrimp. In the present study, an eight-week feeding experiment was performed to investigate the growth performance, antioxidant and anti-inflammatory status, metabolism, and mid-intestine morphology of *L. vannamei* fed with and without dietary *Klebsormidium* sp. The study results might provide a reference for formulating the diet of *L. vannamei*.

## Materials and Methods

### Microalgae Culture and Experiment Feeds Preparation

Strains of *Klebsormidium* sp. were obtained from the Culture Collection of Algae at the University of Göttingen (SAG) and scaled up in our laboratory in the following manner: Briefly, a 160-L vertical flat-plate glass photobioreactor and a BG-11 medium (23) with initial 9 mmol L^−1^ nitrogen (nitrogen source: sodium nitrate) and 1% bubbled CO_2_ (v/v) were used to culture *Klebsormidium* sp. Continuous illumination (24 h) with 300 μmol photons m^−2^ s^−1^ was provided for cultivating the *Klebsormidium* sp. for 15 days.

*Klebsormidium* sp. contained 17.97% crude protein, 31.95% crude lipid (including 9.23% LA), and 29.31% carbohydrate (dry matter) (Table 1).

**Table 1:** Main fatty acid profiles (%, dry matter) and proximal compositions (%, dry matter) of *Klebsormidium* sp.

**Items**	***Klebsormidium* sp**.
**Fatty acid profiles (%, dry matter)**	
C16:0	6.94%
C18:0	1.10%
C18:2	9.23%
C18:3	0.74%
others	1.75%
**Proximal composition (%, dry matter)**	
Protein	17.97%
Lipid	31.95%
Carbohydrate	29.31%

As shown in Table 2, two artificial diets (containing approximately 40% crude protein and 7% crude lipid) with 0% (D1) and 5% (D2) *Klebsormidium* sp., respectively, were prepared using the method detailed in our earlier study (24). Briefly, dry ingredients were weighed and mixed thoroughly in a Hobart-type mixer (A-200T, Canada). Then, pre-weighed fish oil, soybean lecithin, soybean oil, and distilled water (35%, v/w) were added to the mixture until a homogenous mixture was obtained. Then, the wet dough was passed through a mono screw extruder (South China University of Technology, China) with a 1.2 mm diameter die. Diets were then dried and stored at −20°C until use.

**Table 2:** Ingredients and proximate compositions of two experimental diets (%, dry matter).

**Ingredients**	**D1**	**D2**
Fish meal	25	25
Soybean meal	27	27
Peanut meal	12	12
Wheat flour	23.4	18.4
Beer yeast	3	3
Shrimp bran powder	3	3
Fish oil	1	1
Soybean lecithin	1	1
Soybean oil	1	1
Choline chloride (50%)	0.5	0.5
Vitamin C phosphate	0.1	0.1
Vitamin and mineral premix^a^	2	2
Monocalcium phosphate	1	1
*Klebsormidium* sp.	0	5
Sum	100	100
**Nutrient levels**^b^ **(%, dry matter)**		
Moisture	7.45	7.62
Crude lipid	7.12	7.25
Crude protein	40.52	40.37

a *Compositions of vitamin and mineral mixture (kg^−1^ of mixture): vitamin A, 250,000 IU; riboflavin, 750 mg; pyridoxine HCl, 500 mg; cyanocobalamin, 1 mg; thiamin, 500 mg; menadione, 250 mg; folic acid, 125 mg; biotin, 10 mg; a-tocopherol, 3,750 mg; myo-inositol, 2,500 mg; calcium pantothenate, 1,250 mg; nicotinic acid, 2,000 mg; vitamin D_3_, 45,000 IU; vitamin C, 7,000 mg, Zn, 4,000 mg; K, 22.500 mg; I, 200 mg; NaCl, 2.6 g; Cu, 500 mg; Co, 50 mg; FeSO_4_, 200 mg; Mg, 3,000 mg; Se, 10 mg*.

b *Measured values*.

### Experimental Shrimp and Experimental Environment Management

Experimental *L. vannamei* were obtained from the Chinese Academy of Fishery Science (Lingshui, China). Shrimp were fed the D1 group diet to acclimatize them to the experimental environment for 1 month before the feeding trial. Then, after a 24-h starvation treatment, 320 shrimp (initial body weight 0.64 ± 0.02 g) were distributed randomly into the recirculating water system with eight cylindrical fiber tanks (300 L). Each diet was randomly assigned to quadruplicate tanks. The feeding frequency was three times per day at 06:00, 12:00, and 18:00 with 8% of total shrimp weight for 56 days. During the experimental period, environmental conditions were maintained as follows: water temperature: 26.8–28.1°C; pH: 7.5–7.7; salinity: 29–32‰; dissolved oxygen: > 7.0 mg/L; total ammonia nitrogen: < 0.1 mg/L; and sulfide: < 0.05 mg/L.

### Sample Collection

At the end of the feeding trial and after a 24-h starvation treatment, all experimental *L. vannamei* from each tank were weighed and counted. Then, eight shrimp, collected randomly from each tank, were anesthetized (MS-222, Sigma, USA) for obtaining the blood sample. Their hepatopancreas was separated for studying antioxidant parameters and mRNA expression analysis. At the same time, a similar section of the *L. vannamei* mid-intestine was obtained and fixed in 4% paraformaldehyde for intestinal histological examination. Blood samples were stored at 4°C for 12 h and then centrifuged (7,100 g, 10 min, 4°C) to obtain hemolymph. All hepatopancreas and hemolymph samples were separated rapidly and then maintained in liquid nitrogen until examination.

### Diets and *Klebsormidium* sp. Composition Analysis

Moisture, crude lipid, and crude protein of diets and *Klebsormidium* sp. were determined using the standard method of AOAC (25). Briefly, crude protein content (N × 6.25) was determined by the Kjeldahl method (1030- Autoanalyzer; Tecator, Höganäs, Sweden); crude lipid content inspection was performed according to the Soxhlet extractor method (Soxtec System HT6, Tecator, Sweden); moisture content was examined by drying in the ventilated oven at 105°C for 24 h. The carbohydrate content of *Klebsormidium* sp. was analyzed using the modified phenol-sulfuric acid method (5). Analysis of the fatty acid profiles of *Klebsormidium* sp. was done using Agilent Gas Chromatograph (Agilent 6890 N GC, Agilent Technologies, USA) following Zhang et al. (26).

### Hepatopancreas and Hemolymph Antioxidant Parameters Quantification

We followed the method detailed in our previous study for homogenizing hepatopancreas (27). Briefly, hepatopancreas was homogenized (1:9) in phosphate buffer and then the homogenate was centrifuged (10 min, 4°C, 1,200 g). Afterward, the supernatant was collected for further analysis.

Activities of total superoxide dismutase (T-SOD) (A001-1), total antioxidant capacity (T-AOC) (A015–2), glutathione peroxidase (GSH-PX) (A005-1), and the content of malondialdehyde (MDA) (A003-1) were measured following kits' instructions (Nanjing Jiancheng Bioengineering Institute, China) (Kits' instructions can be seen in additional appended files).

### Determination of Mid-Intestine Histological

The section of the mid-intestine was stained according to the study of Zhao et al. (28). Specifically, the mid-intestine section was stained using H&E, and the histological was observed under the microscope (Olympus CKX41 microscope, Japan).

### MRNA Isolation and Expression Quantification

Hepatopancreas RNA isolation and expression quantification were performed according to the method detailed in our previous study (29). Briefly, total RNA was isolated using Trizol® reagent (Invitrogen, USA) following the manufacturer's instructions. To ascertain RNA quality and quantity, we used 1% agarose gel electrophoresis and a spectrophotometer, respectively (NanoDrop 2000, Thermo Fisher, United States). Afterward, cDNA was synthesized using the PrimeScript TM RT reagent Kit (Takara, Japan), following the manufacturer's instructions. Real-time PCR for target genes was performed using an SYBR® Premix Ex TaqTM II (Takara, Japan) and quantified on the LightCycler 480 (Roche Applied Science, Basel, Switzerland). We used *ef1a* as the housekeeping gene for gene expression in the present study (30). Relative mRNA expression levels of target genes were determined using the 2^−ΔΔCT^ method (31). Primers related to the present study are shown in Table 3.

**Table 3:** Sequences of primers used for real-time quantitative PCR.

**Gene**	**Primer sequence (5^**′**^–3^**′**^)**	**Reference**
*ef1a*-F	TGGCTGTGAACAAGATGGAC	(32)
*ef1a*-R	AGATGGGGATGATTGGGACC	
*sod*-F	CCGTGCAGATTACGTGAAGG	(33)
*sod*-R	GTCGCCACGAGAAGTCAATG	
*gsh-px*-F	GGCACCAGGAGAACACTAC	(32)
*gsh-px*-R	CGACTTTGCCGAACATAAC	
*cat*-F	TACTGCAAGTTCCATTACAAGACG	(34)
*cat*-R	GTAATTCTTTGGATTGCGGTCA	
*relish*-F	CTACATTCTGCCCTTGACTCTGG	(32)
*relish*-R	GGCTGGCAAGTCGTTCTCG	
*rho*-F	GTGATGGTGCCTGTGGTAAA	(32)
*rho*-R	GCCTCAATCTGTCATAGTCCTC	
*hsp70*-F	CAACGATTCTCAGCGTCAGG	(33)
*hsp70*-R	ACCTTCTTGTCGAGGCCGTA	
*chymotrypsin*-F	GGCTCTCTTCATCGACG	(35)
*chymotrypsin*-R	CGTGAGTGAAGAAGTCGG	
*trypsin*-F	TCCAAGATCATCCAACACGA	(35)
*trypsin*-R	GACCCTGAGCGGGAATATC	
*hk*-F	AGTCGCAGCAACAGGAAGTT	(36)
*hk*-R	CGCTCTTCTGGCACATGATA	
*fas*-F	GCGTGATAACTGGGTGTCCT	(36)
*fas*-R	ACGTGTGGGTTATGGTGGAT	

### Statistical Analysis

All data in our present study are shown as means ± standard error (SE). Data analysis was performed in SPSS 22.0 (SPSS, Chicago, IL, USA), followed by an independent sample *t-*test where *p* < 0.05 was regarded as the significant difference between groups.

## Results

### Growth Performance

As shown in Table 4, the diet supplemented with *Klebsormidium* sp. significantly improved the growth performance and feed utilization of *L. vannamei*. Substantially higher growth performance parameters [weight gain rate (WGR) and specific growth rate (SGR)] of *L. vannamei* were obtained in the D2 group than in the D1 group (*p* < 0.05). The feed conversion ratio (FCR) of *L. vannamei* fed with *Klebsormidium* sp. was significantly lower compared to the control group (*p* < 0.05). After the 8-week diet treatment, the survival rate (SR) of *L. vannamei* ranged from 96.25 to 99.38% in the present study (*p* > *0.05*).

**Table 4:** Growth performance and feed utilization of *Litopenaeus vannamei* fed diets supplemented with/without *Klebsormidium* sp. for 56 days.

	**D1**	**D2**
IBW	0.64 ± 0.01	0.64 ± 0.02
FBW	5.98 ± 0.03	6.26 ± 0.05
WGR	828.31 ± 15.07	935.4 ± 6.71
SGR	3.98 ± 0.03	4.17 ± 0.01
FCR	1.24 ± 0.03	1.13 ± 0.01
SR	96.25 ± 1.25	99.38 ± 0.63

*IBW (g per shrimp): initial body weight*.

*FBW (g per shrimp): final body weight*.

*Weight gain rate (WGR, %) = 100 × (final body weight – initial body weight)/initial body weight*.

*Specific growth rate (SGR, % day^−1^): 100 × (Ln final shrimp weight – Ln initial shrimp weight)/the experimental duration in days*.

*Feed conversion ratio (FCR) = dry diet fed/wet weight gain*.

*Survival rate (SR) (%) = 100 × (final number of shrimp)/(initial number of shrimp)*.

*Values are mean ± SE (n = 4). Means in the same row with different superscripts are significantly different (p < 0.05)*.

### Antioxidant Capacity

The antioxidant parameters of *L. vannamei* fed with and without *Klebsormidium* sp. are shown in Table 5. *L. vannamei* fed with *Klebsormidium* sp. showed significantly lower antioxidant enzyme activities (hepatopancreas T-SOD, hepatopancreas GSH-PX, and hemolymph T-SOD) compared to the control group (*p*< *0.05*). In addition, significantly lower hemolymph MDA content was found in the dietary *Klebsormidium* sp. treatment group than in the control group (*p*< *0.05*). No statistical differences in hepatopancreas T-AOC, hepatopancreas MDA content, hemolymph T-AOC, and hemolymph GSH-PX were found between the two experimental groups (*p* > *0.05*).

**Table 5:** Hepatopancreas and hemolymph antioxidant parameters of *L. vannamei* fed diets supplemented with/without *Klebsormidium* sp. for 56 days.

	**D1**	**D2**
**Hepatopancreas**		
T-SOD (U/mgprot)	10.4 ± 0.88^a^	7.11 ± 0.68^b^
T-AOC (U/mgprot)	0.27 ± 0.01	0.2 ± 0.04
GSH-PX (U/mg prot)	624.12 ± 49.36^a^	201.49 ± 72.37^b^
MDA (nmol/mgprot)	1.26 ± 0.03	1.02 ± 0.02
**Hemolymph**		
T-SOD (U/mL)	273.75 ± 6.08^a^	219.35 ± 12.54^b^
T-AOC (U/mL)	3.7 ± 0.12	2.78 ± 0.19
GSH-PX (U/mL)	419.35 ± 54.11	522.58 ± 49.27
MDA (mmol/ mL)	8.27 ± 1.04^a^	5.38 ± 1.15^b^

*Values are mean ± SE (n = 4). Means in the same row with different superscripts are significantly different (p < 0.05)*.

**Figure 1:**
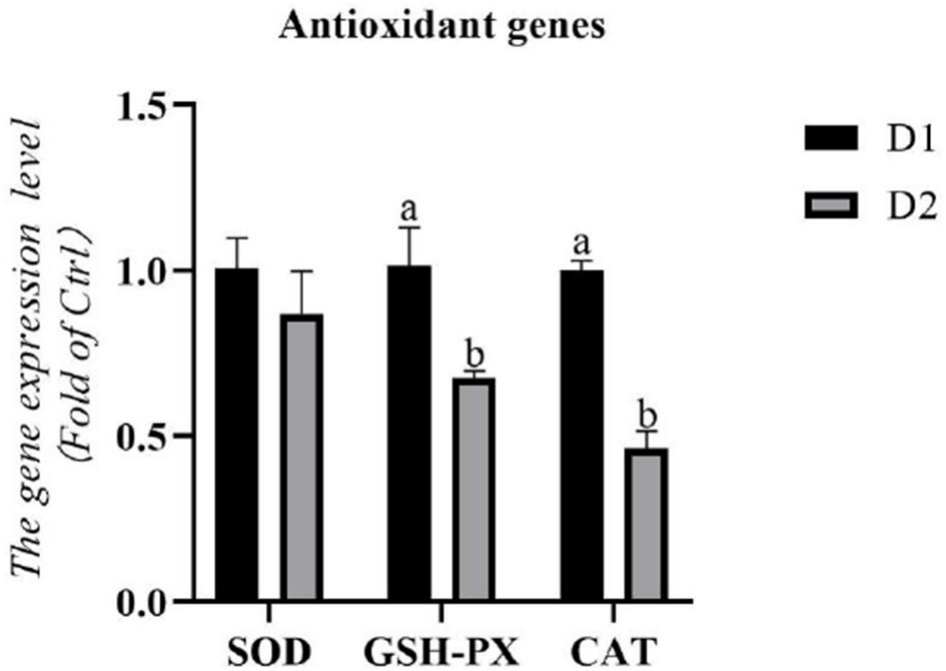
Hepatopancreas RNA expression levels of antioxidant genes of *Litopenaeus vannamei* fed diets supplemented with/without *Klebsormidium sp*. for 56 days. ^*a, b*^The small letters indicated significant differences at *p* < 0.05.

The RNA expression levels of antioxidant genes in the hepatopancreas are shown in Figure 1. The RNA expression levels of *catalase* (*cat*) and *gsh-px* in the D2 group were significantly lower than that in the D1 group (*p*< *0.05*). However, dietary *Klebsormidium* sp. could not alter the RNA expression level of *sod* in the hepatopancreas (*p* > *0.05*).

### RNA Expression Levels Related to Pro-Inflammatory Genes

**Figure 2:**
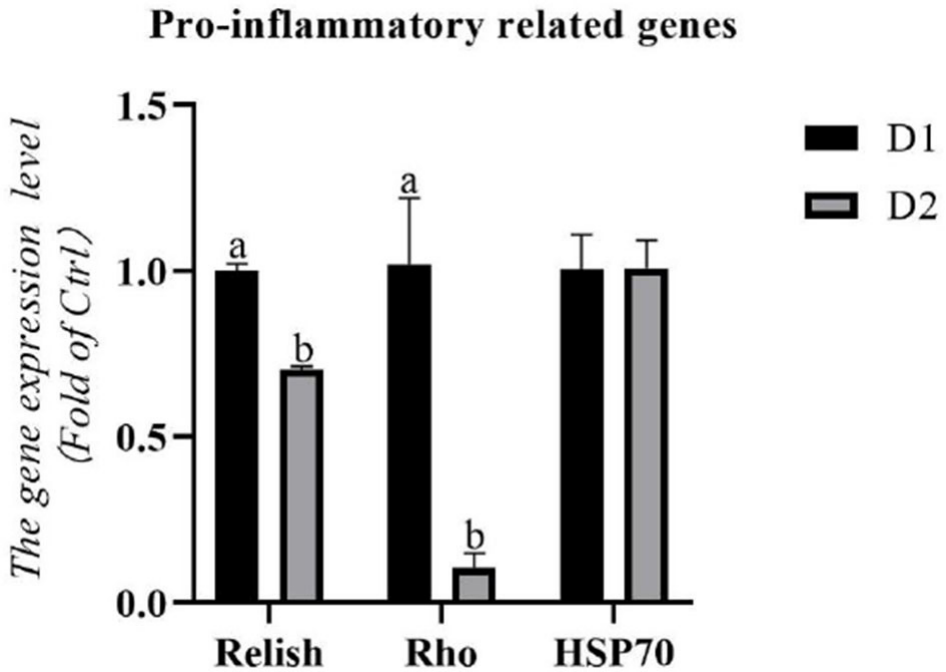
Hepatopancreas RNA expression levels of pro-inflammatory related genes of *L. vannamei* fed diets supplemented with/without *Klebsormidium sp*. for 56 days. ^*a, b*^The small letters indicated significant differences at *p* < 0.05.

The RNA expression levels of pro-inflammatory related genes in the hepatopancreas are shown in Figure 2. Remarkably lower RNA expression levels of pro-inflammatory related genes (*relish* and *rho*) were obtained in the D2 group than that in the D1 group (*p*< *0.05*). There was no significant difference in the RNA expression level of *hsp70* between the experimental groups (*p* > *0.05*).

### RNA Expression Levels Related to Digestion and Metabolism

**Figure 3:**
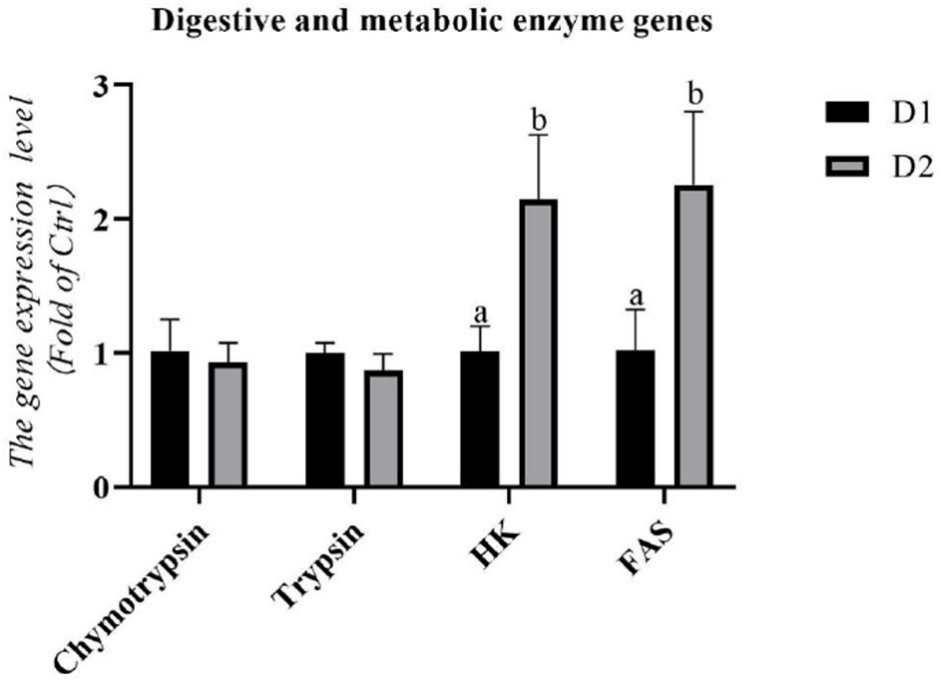
Hepatopancreas RNA expression levels of digestive and metabolic enzyme genes of *L. vannamei* fed diets supplemented with/without *Klebsormidium sp*. for 56 days. ^*a, b*^The small letters indicated significant differences at *p* < 0.05.

As shown in Figure 3, the diet supplemented with *Klebsormidium* sp. could not alter the RNA expression levels of digestive enzyme genes (*chymotrypsin* and *trypsin*) in the hepatopancreas of *L. vannamei* (*p* > *0.05*).

The RNA expression levels of metabolic enzyme genes in the hepatopancreas are shown in Figure 3. Significantly higher mRNA expression of *hexokinase* (*hk*) and *fatty acid synthase* (*fas*) were found in the D2 group compared to the D1 group (*p*< *0.05*).

### Mid-Intestine Morphology

**Figure 4:**
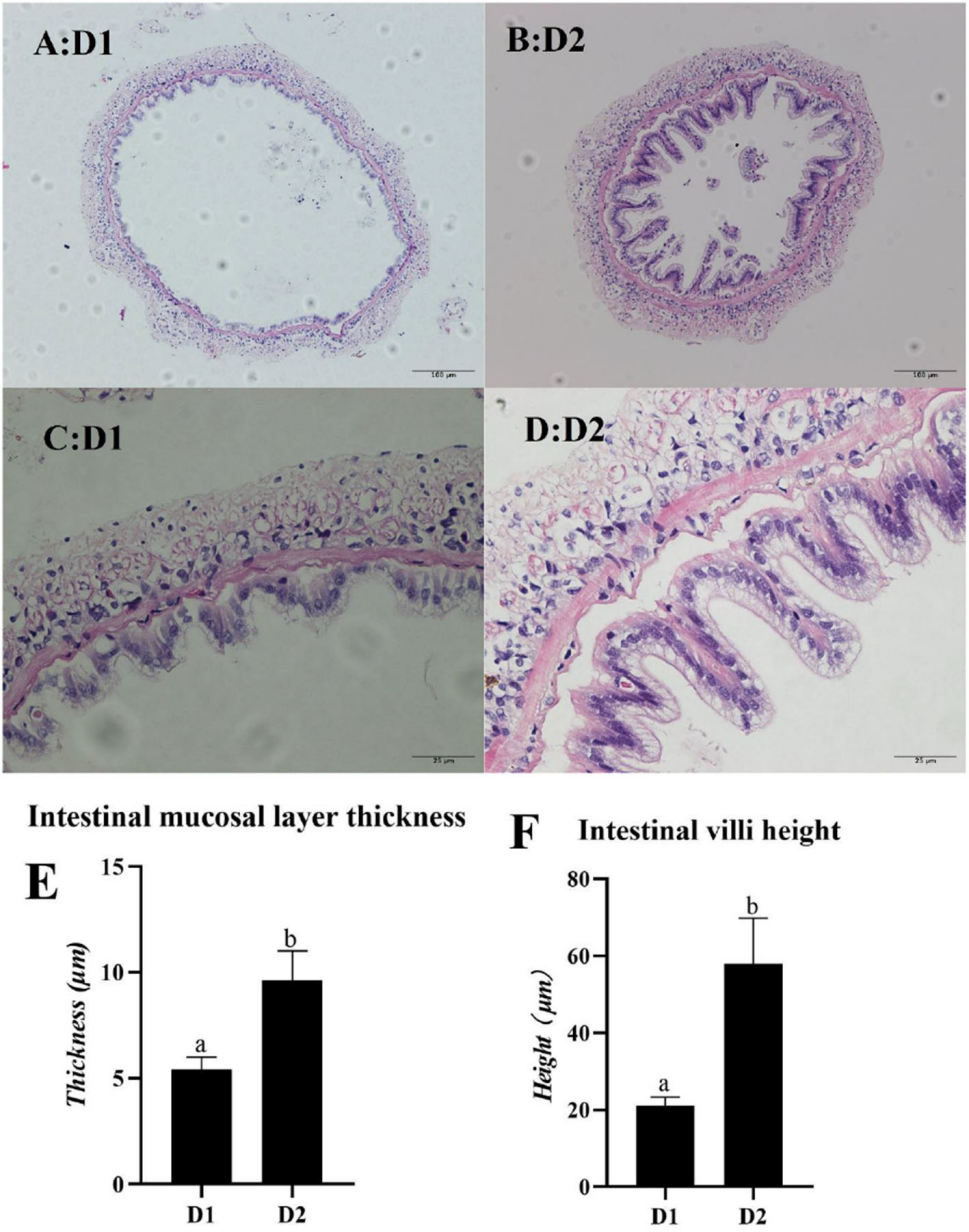
Light microscopy of mid-intestine morphology of *L. vannamei* fed diets supplemented with/without *Klebsormidium sp*. for 56 days. Scale bars **(A,B)** = 100 μm; Scale bars **(C,D)** = 25 μm. **(E,F)** represent the mid-intestinal mucosal layer thickness and intestinal villi height of *L. vannamei*. ^*a, b*^The small letters indicated significant differences at *p* < 0.05.

The mid-intestine morphology of *L. vannamei* fed with and without *Klebsormidium* sp. is shown in Figure 4. Mid-intestine morphology parameters (the intestinal mucosal layer thickness and the intestinal villi height) in the D2 group were significantly higher than that of the D1 group (*p*< *0.05*).

## Discussion

As a green additive in aquatic feed, microalgae have gained more attention in recent years due to their high nutritional value and convenience of scaling up the culture (37). Different microalgae might contain various nutrients, such as polyunsaturated fatty acid (PUFAs) (38, 39), pigment (40, 41), vitamins (42), minerals (43), and algae polysaccharides (44), which are beneficial to the health of aquatic animals. Therefore, microalgae have huge potential to substitute synthetic additives and reduce the budget of the aquatic feed in aquaculture (45).

In the present study, *L. vannamei* fed with *Klebsormidium* sp. obtained better growth performance parameters (WGR and SGR) and feed utilization (FCR) than the control group. These results are similar to the studies of *L. vannamei* fed with *Haematococcus pluvialis* (46), *Trachinotus ovatus* fed with *Tribonema* sp. (28), and *Oplegnathus fasciatus* fed with *Spirulina* (47). However, no significant difference in growth performance of *Carassius auratus gibelio* fed with/without *Tribonema* sp. was reported in the study of Chen et al. (48). Different results in previous studies might be attributed to differences in animal species, animal size, microalgae species, the dose of dietary microalgae, and the experimental condition. The degree of development of the intestinal morphology was one of the essential indices to affect the growth performance and feed utilization of animals because the gut contacted and absorbed the nutrients directly (49). In particular, higher intestinal villi height indicated a larger contact area between the gut and nutrients and combined with a thicker intestinal mucosal layer, it implied better absorption ability of animals (50, 51). The present study demonstrated that dietary *Klebsormidium* sp. supplements were significantly beneficial to mid-intestine morphology parameters (the intestinal villi height and the intestinal mucosal layer thickness) of *L. vannamei*. A previous study showed that a diet supplemented with fish oil (EPA, DHA-rich) or perilla oil (ALA-rich) had protective effects on the intestine morphology of rat samples since n-3 PUFA could mitigate the TNBS-induced colitis, thus reducing the death of intestinal epithelial cells (52, 53). You et al. (54) and other researchers (28, 55, 56) also pointed out that dietary n-3 PUFAs are beneficial to the gut morphology development of aquatic animals. In the present study, better mid-intestine morphology of *L. vannamei* was obtained in the D2 group, which might be because of rich LA in *Klebsormidium* sp. that could be converted to EPA and DHA (57), resulting in effective growth performance and feed utilization of *L. vannamei*.

The growth performance of *L. vannamei* is also influenced by its metabolic capacity because better metabolic capacity indicates a better nutrient utilization ability in shrimp (58). In addition, hexokinase could convert D-hexose into D-hexose-6-phosphate, which is one of the most important rate-limiting steps in the glycolysis reaction (59). In the present study, the mRNA expression level of *hk* was significantly upregulated in *L. vannamei* fed with *Klebsormidium* sp. Layam and Reddy (60) had shown that dietary *Spirulina* increased the hexokinase activity of streptozotocin-diabetic rats. However, few studies have been published about the regulating mechanism of microalgae on glycolysis. A previous study demonstrated that conjugated linoleic acid could activate the AMPK pathway in chick embryos (61), an essential pathway associated with regulating cellular energy homeostasis. Therefore, in the present study, the upregulation of the mRNA expression level of *hk* might be attributed to the LA in *Klebsormidium* sp., activating the AMPK pathway and then regulating the glycolysis of *L. vannamei*. Without a doubt, further study of this subject is required. In the present study, the improvement of carbohydrate metabolism capacity may contribute to improving the growth performance and feed utilization of *L. vannamei*. In addition, other micronutrients like carotenoids (62) in *Klebsormidium* sp. might be attributed to promoting the growth.

Respiratory bursts will occur if aquatic animals are subjected to environmental stressors, which could produce reactive oxygen species (ROS) for reducing oxidative stress (63). This is one of the effective self-defense mechanisms in cells. However, overproduction of ROS might also attack normal physiological cells and thus cause oxidative damage to cells (64). In this situation, cells would activate the antioxidant system and enhance the activity of antioxidant enzymes (like SOD, GSH-PX, CAT) to scavenge the overproduction of ROS for protecting the cells from oxidative stressors (65, 66). Therefore, the activity of antioxidant enzymes could be regarded as an important index to evaluate the antioxidant capacity of *L. vannamei*. Except for antioxidant enzymes, the MDA content, which reflects the damage degree of cell structure, can also be used to assess the oxidative state of shrimp (64, 67). In the present study, significantly lower antioxidant enzyme activities (hepatopancreas T-SOD, hepatopancreas GSH-PX, and hemolymph T-SOD), RNA expression levels of hepatopancreas antioxidant genes (*gsh-px* and *cat*), and hemolymph MDA content were obtained in the dietary *Klebsormidium* sp. treating group compared to the control group. Previous studies have shown that PUFAs-rich microalgae (such as *Nannochloropsis, Tetraselmis*, and *Thalassiosira*) could bring various health benefits (68, 69). Besides, the inhibition effect of free radical-induced DNA break by LA has been demonstrated in *in vitro* studies, indicating its potential medicinal value (70). Therefore, in the present study, a diet supplemented with *Klebsormidium* sp. could improve the antioxidant capacity of *L. vannamei*, which was correlated to the LA.

Apart from the respiratory burst, an inflammatory response is another important self-defense mechanism of cells for eliminating pathogens (71). However, an excessive inflammatory response might attack healthy tissues and cells and cause various pathological diseases (72). Among them, the NF-κB pathway was one of the crucial inflammatory responses of *L. vannamei* (32). *Relish* and *rho* are two well-known transcription factors involved in the NF-κB pathway (32, 73). In the present study, *L. vannamei* fed with *Klebsormidium* sp. obtained remarkably lower RNA expression levels of *relish* and *rho* than the control group, indicating that *Klebsormidium* sp. could mitigate the pro-inflammatory response. This result might be due to the rich LA in *Klebsormidium* sp. Previous studies showed that n-3 PUFA (EPA, DHA) could inhibit the inflammatory response of rodent samples by reducing the inflammatory mediators (like prostaglandin E2 (PGE2), leukotriene B4 (LTB4), TNF-α, and IL-6) (74–76). Therefore, *Klebsormidium* sp. could improve the anti-inflammatory capacity of *L. vannamei* by suppressing the NF-κB pathway.

## Conclusion

Overall, diet supplementations of 5% *Klebsormidium* sp. were beneficial to *L. vannamei* since they could improve the growth performance, antioxidant and anti-inflammatory status, carbohydrate metabolism, and mid-intestine morphology of shrimp. Therefore, the dose of 5% *Klebsormidium* sp. is recommended for the daily diet of *L. vannamei*.

## Data Availability Statement

The raw data supporting the conclusions of this article will be made available by the authors, without undue reservation.

## Ethics Statement

The animal study was reviewed and approved by the Experimental Animal Ethics Committee of Sun Yat-sen University.

## Author Contributions

HF, JN, and WZ designed the study. LH cultured the *Klebsormidium* sp. HF and ZZ analyzed data. HF carried out the investigation and wrote this paper. WZ modified the language. All authors contributed to the article and approved the submitted version.

## Funding

This work was supported by Project of Science and Technology of Guangdong Province (2021B0202050002), Project of China Agriculture Research System of MOF and MARA 48 (CARS 48), Project of Science and Technology of Guangdong Province (2019B110209005), and Youth Science, and Technology Innovation Talent of Guangdong TeZhi Plan Talent (2019TQ05N129).

## Conflict of Interest

The authors declare that the research was conducted in the absence of any commercial or financial relationships that could be construed as a potential conflict of interest.

## Publisher's Note

All claims expressed in this article are solely those of the authors and do not necessarily represent those of their affiliated organizations, or those of the publisher, the editors and the reviewers. Any product that may be evaluated in this article, or claim that may be made by its manufacturer, is not guaranteed or endorsed by the publisher.
