# Supplementary material for: Dietary Klebsormidium sp. Supplementation Improves Growth Performance, Antioxidant and Anti-Inflammatory Status, Metabolism, and Mid-Intestine Morphology of Litopenaeus Vannamei
Source: Front Nutr. 2022 May 12;9:857351. doi: 10.3389/fnut.2022.857351 (PMC9136981; doi:10.3389/fnut.2022.857351)
Supplement: Supplementary file 1 [file Data_Sheet_1.pdf]

Superoxide Dismutase（SOD）Assay Kit

(Hydroxylamine Method)

Serial Number：A001-1 Pack: 100T/96S 50T/48S

Catalogue

Superoxide Dismutase (SOD) Assay Kit ········ **Error! Bookmark not defined.**
Serial Number：A001-1 ········ **Error! Bookmark not defined.**
Catalogue ········ **Error! Bookmark not defined.**
Introduction of assay kit ········ **Error! Bookmark not defined.**
Assay significance ········ **Error! Bookmark not defined.**
Kit expiry date and storage condition ········ **Error! Bookmark not defined.**
Required equipments and reagents in experiment ········ 3
Reagents' composition and preparation ········ **Error! Bookmark not defined.**
Total superoxide dismutase (T-SOD) activity assay ········ 5
Optimal sample volume probing ········ 7
Reference values ········ 8
Announcements ········ 9
Advantages of this Kit ········ 11
Appendix: Experimental Methodology ········ 12
Appendix I: SOD standard curve preparation ········ 13
Appendix II: T-SOD assay in blood serum (or plasma) and related liquids ········ 14
Appendix III: Animal tissue SOD assay ········ 15
Appendix IV: T-SOD assay in hyperlipemia blood serum (or plasma) ········ 18
Appendix V: Erythrocyte SOD assay ········ 20
Appendix VI: Plant tissue SOD assay ········ 22

Address:Central Road, Xuanwu District, Nanjing Xinliji House 258-27 Postal code:210009 Fax:025-83227943
E-Mail:njjcbio@vip.163.com Tel:025-83360321、83360969、83551389 Technology:025-83360272

Introduction of SOD assay kit

Xanthine Oxidase method is used in this kit to measure superoxide dismutase (SOD) activity. This kit applies to measure SOD activities in blood serum (or plasma), cerebrospinal fluid, hydrothorax, hydroperitoneum, kidney dialysate, urine, erythrocytes, leucocytes, blood platelets, cardiac muscle cultured cells, tumor cultured cells, various kinds of animal or plant tissue cells and subcellsular level (mitochondria, microsome), it also can measure SOD activities in microbes, medicines, foods, drinks, cosmetics.

Assay significance

Superoxide dismutase (SOD) plays an important role in oxidation-antioxidation balance in organisms, this enzyme can remove superoxide anion radicals ($O_2^{-}\cdot$) to protect cells away from damage.

Assay principle

Use xanthine and xanthine oxidase reaction system to produce superoxide anion radicals ($O_2^{-}\cdot$), the latter will oxidate hydroxylamine to form nitrite, appears prunosus color under effect of chromogenic agent, its absorbance can be measured by visible range spectrophotometer. If sample to assay contains SOD, then it has a narrow spectrum depressant effect for superoxide anion radicals, as the result, absorbance in sample tube will be lower than absorbance in contrast tube, SOD activity can be calculated by formula.

There are only 2 types of SOD in higher animal cells, they are CuZn-SOD and Mn-SOD, lesser animals/ unicellular animals/plants have Fe-SOD besides CuZn-SOD & Mn-SOD. It is able to calculate SOD activities of all types according to measured values.

Kit expiry date and storage condition

This kit's expiry date: 6 months; storage temperature : 4℃.

Note: Reagent 4 can not be stored at temperature below 0℃ or enzyme will loss activity， plastic tips used in assay must be cleansed (disinfection is even better, or wash new plastic tips repeatedly with distilled water), bacteria pollution and heavy metal ion pollution must be avoided.

Address:Central Road, Xuanwu District, Nanjing Xinliji House 258-27 Postal code:210009 Fax:025-83227943
E-Mail:njjcbio@vip.163.com Tel:025-83360321、83360969、83551389 Technology:025-83360272

Required equipments and reagents in experiment

1. 550nm spectrophotometer
2. 37℃ thermostatic water bath
3. Desk centrifuge
4. Micropippet
5. Double distilled water
6. Glacial acetic acid (analytical pure, acetic acid concentration ≥99.5%)

Reagents' composition and preparation

1. 100T kit's composition and preparation: (100T/96S)

Reagent 1: **Stock solution:** 10ml×1 bottle (crystals may seed out at low temperature or in fridge, so it needs hot water bath to dissolve before use.);

Reagent 1 working solution preparation: dilute each bottle of stock solution (10ml) with double distilled water until mixture volume reaches 100ml, can be stored at 4℃ for 1 year.

Reagent 2: Liquid, 10ml×1 bottle, can be stored at 4℃~10℃ for 1 year.

Reagent 3: Liquid, 10ml×1 bottle, can be stored at 4℃~10℃ for 1 year.

Reagent 4: **Stock solution,** 350μl×2 vials, **can be stored at -20℃**;

Diluent: 10ml×1 bottle, can be stored at 4℃ for 6 months.

Reagent 4 working solution preparation: Dilute Reagent 4 with diluent at ratio of 1:14, **consider prepared volume according to your need. Working solution can be stored at 4℃ (No freezing!).**

Note: please use disposable tips.

Reagent 5: Powder ×1 tube, add 75ml double distilled water at 70℃~80℃, dissolve powder before use. If water volume reduces by evaporation in heating process, then add more double distilled water to make volume reaches 75ml. Dispensed solution can be stored **at 4℃ away from light for 1 year.**

Reagent 6: Powder×1 tube, add 75ml double distilled water to dissolve before use. Prepared solution can be stored **at 4℃ away from light for 3 months.**

Chromogenic agent preparation: Mix Reagent 5, Reagent 6 and glacial acetic acid at volume ratio of 3:3:2, prepared chromogenic agent can be stored at 4℃ away from light for 3 months.

Note: Glacial acetic acid (analytical pure, acetic acid ≥99.5%)

Please prepare Reagent 5, Reagent 6 separately (do not mix Reagent 5 and Reagent 6 together before preparation or chromogenic agent won't work).

Address:Central Road, Xuanwu District, Nanjing Xinliji House 258-27 Postal code:210009 Fax:025-83227943
E-Mail:njjcbio@vip.163.com Tel:025-83360321、83360969、83551389 Technology:025-83360272

2. 50T kit's composition and preparation (50T/48S):

Reagent 1: **Stock solution:** 5ml×1 bottle (crystals may seed out at low temperature or in fridge, so it needs hot water bath to dissolve before use.);

Reagent 1 working solution preparation: Dilute each bottle of stock solution (10ml) with distilled water until mixture volume reaches 10ml, working solution can be stored at 4℃ for 1 year.

Reagent 2: Liquid, 5ml×1 bottle, can be stored at 4℃~10℃ for 1 year.

Reagent 3: Liquid, 1ml×1 bottle, can be stored at 4℃~10℃ for 1 year.

Reagent 4: **Stock solution,** 350μl×1 vial, **can be stored at -20℃**;

Diluent: 5ml×1 bottle, can be stored at 4℃ for 6 months.

Reagent 4 working solution preparation: Dilute Reagent 4 with diluent at ratio of 1:14, **consider prepared volume according to your need. Working solution can be stored at 4℃ (No freezing!).**

Note: please use disposable tips.

Reagent 5: Powder×1 tube, add 37.5ml double distilled water at 70℃~80℃, dissolve powder before use. If water volume reduces by evaporation in heating process, then add more distilled water to make volume reaches 37.5ml. Dispensed solution can be stored **at 4℃ away from light for 6 months.**

Reagent 6: Powder×1 tube, add 37.5ml distilled water to dissolve before use. Prepared solution can be stored **at 4℃ away from light for 3 months.**

Chromogenic agent preparation: Mix Reagent 5, Reagent 6 and glacial acetic acid at volume ratio of 3:3:2, prepared chromogenic agent can be stored at 4℃ away from light for 3 months.

Note: Glacial acetic acid (analytical pure, acetic acid ≥99.5%)

Please prepare Reagent 5, Reagent 6 separately (do not mix Reagent 5 and Reagent 6 together before preparation or chromogenic agent won't work).

Address:Central Road, Xuanwu District, Nanjing Xinliji House 258-27 Postal code:210009 Fax:025-83227943
E-Mail:njjcbio@vip.163.com Tel:025-83360321、83360969、83551389 Technology:025-83360272

Total Superoxide Dismutase (T-SOD) Activity Assay

1. Operation table:

Total superoxide dismutase（T-SOD）activity assay:

Reagents	Sample tube	Contrast tube
Reagent 1 working solution (ml)	1.0	1.0
Sample(ml)	a*	
Double distilled water (ml)		a*
Reagent 2(ml)	0.1	0.1
Reagent 3(ml)	0.1	0.1
Reagent 4(ml)	0.1	0.1
**Mix sufficiently by vortex，place in 37℃ thermostatic water bath for 40 minutes**		
Chromogenic agent (ml)	2	2
Mix sufficiently, place at room temperature for 10 minutes, use spectrophotometer to measure absorbances at 550nm by 1cm light path glass cuvette (adjust zero by double distilled water).,		

2. Calculations:

(1) Calcultions of total SOD activities in blood serum (or plasma), cadiac muscle perfusate, kidney dialysate, cell culture fluid, etc :

a. Definition: Corresponding quantity of SOD that its inhibition ratio pecentage reaches to 50% per ml reaction solution is considered as one SOD activity unit (U).

b. Formula of total SOD activity in blood serum (or plasma), cardiac muscle perfusate, kidney dialysate, cell culture fluid, etc.:

$$\text{Total SOD activity (U/ml)} = \frac{A_{Contrast} - A_{Sample}}{A_{Contrast}} \div 50\% \times \text{Reaction system dilution times} \times \text{Sample dilution times before assay}$$

$A_{Contrast}$ is absorbance in contrast tube, A_{Sample} is absorbance in sample tube.

(2) Calculations of total SOD activity in animal tissue homogenate:

a. Definition: Corresponding quantity of SOD that its inhibition ratio pecentage reaches to 50% per mg tissue protein in 1ml reaction solution is considered as one SOD activity unit (U).

b. Calculating formula:

$$\text{T-SOD activity in tissue homogenate (U/mgprot)} = \frac{A_{Contrast} - A_{Sample}}{A_{Contrast}} \div 50\% \times \frac{\text{Total volume of reaction solution(ml)}}{\text{Sample volume (ml)}} \div \text{Tissue protein concentration (mgprot/ml)}$$

* mgprot means "milligram protein".

(3) Calculations of total SOD activity in plant tissue homogenate:

Method 1: Calculate according to tissue wet weight

Definition: Corresponding quantity of SOD that its inhibition ratio pecentage reaches to 50% per g tissue in 1ml reaction solution is considered as one SOD activity unit (U).

Formula:

$$\text{T-SOD activity (U/g wet tissue)} = \frac{A_{Contrast} - A_{Sample}}{A_{Contrast}} \div 50\% \times \frac{\text{Total volume of reaction solution(ml)}}{\text{Sample volume (ml)}} \div \text{Homogenate concentration (g/ml)}$$

Note:

$$\text{Homogenate concentration (g/ml)} = \frac{\text{Tissue wet weight (g)}}{\text{Homogenate volume (ml)}}$$

Method 2: Calculate according to protein concentration

Definition: Corresponding quantity of SOD that its inhibition ratio pecentage reaches to 50% per mg tissue protein in 1ml reaction solution is considered as one SOD activity unit (U).

Formula:

$$\text{T-SOD activity (U/mgprot)} = \frac{A_{Contrast} - A_{Sample}}{A_{Contrast}} \div 50\% \times \frac{\text{Total volume of reaction solution(ml)}}{\text{Sample volume (ml)}} \div \text{Tissue protein concentration (mgprot/ml)}$$

* mgprot means milligram protein

Announcements

Note 1: a* refers to sample volumes of samples and double distilled water.

Note 2: Optimal sample volumes are various because different kinds of samples have different SOD activities. According to the fact that enzyme's inhibition ratio and enzyme activity appear parabolic relation（appendix: SOD standard curve）, diifferent samples to assay have different sample volumes, it' s suggested to choose an optimal sample volume before assay a new kind of sample.

Note 3: Add reagents in in proper order according to operation table. You can make mixed

Address:Central Road, Xuanwu District, Nanjing Xinliji House 258-27 Postal code:210009 Fax:025-83227943
E-Mail:njjcbio@vip.163.com Tel:025-83360321、83360969、83551389 Technology:025-83360272

reagent of Reagent 1/Reagent 2 or Reagent 1/Reagent 3, adding volume of mixed reagent can be up to 1ml, but DO NOT mix Reagent 1/Reagent 2/Reagent 3/Reagent 4 together or it will disturb result.

Optimal sample volume probing

If it is the first time for you to use this kit to assay a new kind of sample, you'd better to make 3 assay tubes of different sample volumes. Consider referenced optimal sample volume as mean value, use mean value, mean value +10μl, mean value -10μl as sample volumes to make 3 sample tubes, do pre-test by 3 sample tubes and 1 contrast tube in order to get optimal sample volume.

Optimal sample volume calculation: $(A_{Contrast} - A_{Sample}) / A_{Contrast}$

Optimal sample range: $(A_{Contrast} - A_{Sample}) / A_{Contrast}$ (equals to inhibition percentage) is between 0.15 and 0.55 (curve appears direct proportion in this range)

Optimal sample volume choice: take the tube which inhibition percentage is between 45% to 50% as optimal sample volume.

Optimal sample volume adjustion: If inhibition percentage is over 60% (curve appears flat in this part), then you need to dilute sample or decrease sample volume and try again. If inhibition percentage is lower than 20%, then you need to increase sample volume and try again.

In this way, it is great helpful for scientific result analysis and t-test; if inhibition ratio percentage is higher than 60% or lower than 10%, then there are no significant differences between different assay groups.

Note 4：Referenced optimal sample volumes:

① Erythrocyte extract: 8.0~10μl generally；

② Rat erythrocyte extract: about 5μl;

③ Human blood plasma (or serum): 30~50μl generally ;

④ Mouse blood plasma (or serum): about 20μl;

⑤ 1% tissue homogenate: 30~50μl;

⑥ Endochylema: 20~50μl;

⑦ Cardiac muscle perfusate or kidney dialysate: 100~200μl;

⑧ Leucocyte suspension: 100~200μl;

⑨ Cell culture fluid: 100~200μl

All samples can be diluted by physiological saline and enlarge sample volume. (**If dilute 1 time, then enlarge volume 1 time).**

Address:Central Road, Xuanwu District, Nanjing Xinliji House 258-27 Postal code:210009 Fax:025-83227943
E-Mail:njjcbio@vip.163.com Tel:025-83360321、83360969、83551389 Technology:025-83360272

Reference Values

○ Mouse

● **Blood serum(or plasma)** T-SOD activity: 110.446±21.325 U/ml（Sample volume: 20μl）

● **Liver tissue** T-SOD activity: 269.274±23.448 U/mgprot（0.25％tissue homogenate, sample volume: 50μl)

● **Brain tissue** T-SOD activity: 108.790±13.494 U/mgprot（1％ tissue homogenate, sample volume: 50μl）

● **Kidney tissue** T-SOD activity: 154.277±15.646 U/mgprot（0.5％ tissue homogenate, sample volume: 50μl）

● **Cardiac muscle tissue** T-SOD activity: 174.330±19.961 U/mgprot（1％ tissue, sample volume: 50μl）

● **Skin tissue** T-SOD activity: 69.01+19.95 U/mgprot（1％ tissue homogenate, sample volume: 50μl）

● **Skeletal muscle** T-SOD activity: 101.717±12.190 U/mgprot（1％ tissue homogenate, sample volume:50μl）

○ Rat

● **Blood serum (or plasma)** T-SOD activity: 262.786±23.240 U/ml（Sample volume: 5μl）

● **Whole blood** T-SOD activity: 21.554±2.116 U/mgHb

● **Liver tissue** T-SOD activity: 214.689±38.803 U/mgprot（0.25％ tissue homogenate, sample volume: 50μl）

● **Kidney tissue** T-SOD activity: 136.825±24.763 U/mgprot（0.5％tissue homogenate, sample volume: 50μl）

● **Intestine tissue** T-SOD activity: 74.738±11.351 U/mgprot（1％ tissue homogenate, sample volume: 50μl）

● **Lung tissue** T-SOD activity: 35.542±15.465 U/mgprot（2％ tissue homogenate, sample volume: 50μl）

● **Brain tissue** T-SOD activity: 140.177±26.878 U/mgprot（1％ tissue homogenate, sample volume: 50μl）

● **Cerebral cortex tissue** T-SOD activity: 79.037±3.996 U/mgprot（1％ tissue homogenate, sample volume: 50μl）

● **Hippocampus tissue** T-SOD activity: 136.863±36.472 U/mgprot（1％ tissue homogenate, sample

volume: 50μl）

● **Cardiac muscle tissue** T-SOD activity: 128.292±9.219 U/mgprot（0.5％ tissue homogenate, sample volume: 50μl）

○ **Rabbit**

● **Blood serum** T-SOD activity: 429.04±31.60U/ml（Sample volume: 10μl）

○ **Chicken**

● **Blood serum** T-SOD activity: 213.208±73.368 U/ml

○ **Bovine**

● **Blood serum** T-SOD activity: 123.691±20.008 U/ml

● **Gastric juice** T-SOD activity: 27.880±8.076 U/ml

○ **Human**

● **Blood serum** T-SOD activity: 104.2±18.8U/ml （n=100 people, sample volume: 30μl);

Mn-SOD activity：41.21± 3.2U/ml (n=100 people, sample volume: 30μl);

● **Erythrocyte** T-SOD activity: 19246±132U/gHb（n=40 people， Erythrocyte extract sample volume: 10μl）;

● **Whole blood** T-SOD activity: 21.554±2.117 U/mgHb

All values above are in the form of $\overline{X}$ ± **SD**

It is suggested to set up normal reference value range of your own laboratory.

Announcements：

(1) If you measure microscale samples such as cultured cardiac muscle cells, cultured tumor cells, leucocytes, blood platelets, mitochondrions, microsome or isolated cardiac muscle perfusate, etc, **then please use disposable plastic test tube or cylindrical micropore plate to operate. You can buy these equipments from our company.**

(2) All reagent preparations should be done 1 day before assay in order to dissolve sufficiently. Prepared reagents can be stored at 4℃ for 3~6 months (except Reagent 4). Please take reagents out of fridgfe and place them at room temperature for 30 minutes before use.

Address:Central Road, Xuanwu District, Nanjing Xinliji House 258-27 Postal code:210009 Fax:025-83227943
E-Mail:njjcbio@vip.163.com Tel:025-83360321、83360969、83551389 Technology:025-83360272

(3) In order to avoid measuring errors, reagent of 1st suction should be discarded, it is used to wash tube. If there is liquid droplets hang outside tip, then use filter paper to wipe them dry softly. Sample and reagents should be added in test tube vertically, do not add on tube surface because volumes of sample and reagents are quite small. Mix sufficiently by vortex before water bath or air bath.

(4) Every incubation time is 40 minutes. If room temperature is lower than 20℃, then please extend incubation time to 45 minutes properly. Incubation temperature should be constant at 37℃.

(5) Make 2 contrast tubes in all samples, take their average value. You can also make 1 contrast tube for every 9 sample tubes.

(6) When you measure T-SOD and Mn-SOD or measure T-SOD and CuZn-SOD or T-SOD and Fe-SOD of a sample, you only need to measure one pair of them. There are only 2 types of SOD (CuZn-SOD and Mn-SOD) in higher animals, CuZn-SOD plus Mn-SOD equals to T-SOD. As result, T-SOD minus Mn-SOD equals to CuZn-SOD, T-SOD minus CuZn-SOD equals to Mn-SOD. Lower animals, unicellular organisms and plants have Fe-SOD, so T-SOD minus Mn-SOD minus equals to Fe-SOD.

(7) Use preliminary experiment to determine optimal sampling volume: If it is the first time for you to use this kit to assay a new kind of sample, you'd better to make 3 sample tubes of different sampling volumes. For example, if you want to measure 1% brain tissue homogenate, then use our quoted sampling volume as intermediate value, increase and decrease sampling volume by 10μl to make 2 other tubes, make 1 contrast tube also. Do preliminary test according to operation table, in order to determine optimal sampling volume. Calculate as follows: $(A_{Contrast}-A_{Sample}) \div A_{Contrast}$ It should be between 0.15 and 0.55, to say in other words, inhibition percentage is between 15% and 55%. Then consider the sampling volume of the tube whose inhibition ratio percentage is 45%~50% as optimal sampling volume. Enzyme's inhibition ratio percentage has parabola relation with enzyme activity, if inhibition ratio percentage is higher than 60%, then please decrease sample concentration by dilution or reduce sampling volume before assay again. If inhibition ratio percentage is lower than 20%, then please increase sampling volume before assay again.

(8) EDTA will combine with heavymetalloenzyme to form chelate. It decreases EDTA activity or even makes negative result. As result, never use EDTA as anticoagulant agent when you collect blood plasma.

Advantages of this kit

1. **Fast:** Whole operation process need about 50 minutes, more than 100 samples can be measured in this period. This short time, large amount assay method is welcome for operators.
2. **Microscale sample volume:** By using this method, it only needs 5μl～10μl peripheral blood from finger or auricular lobule to measure SOD in erythrocytes, only needs 1ml venous blood to measure SOD in leucocytes and platelets, only needs 6mg tissue to measure SOD in tissue homogenate, endochylema, only needs 0.2g tissue to measure SOD in mitochondrions and microsomes, thus this method is welcome for scientific researchers.
3. **High sensitivity:** IC50=0.05g/ml, its sensitivity is 18 times as pyrogallic acid method, so it is most sensitive method to measure SOD in cardiac muscle perfusate, cerebrospinal fluid, hydrothorax, hydroperitoneum, kidney dialysate, cultured cardiac muscle cells, cultured tumor cells.
4. **High stability:** Reagents can be stored in fridge at 0°C～4°C for 6 months. Place pooled serum at 4°C, SOD activity keeps stable in 3 days.
5. **Good repeatability:** Coefficient of variation (CV) = 1.7%, there is no significant deviation between a sample's result this day and this sample's result several days later.
6. **Recovery test:** X±SD=103.3±2.63%
7. **Low influence from outside:** This method was already used by thousands of scientific research units and colleges, they all agree that this method is domestic pantent now, surpasses other chemical assay methods and radio-immunity method.
8. **Wide assay range:** It is able to measure T-SOD, Mn-SOD and CuZn-SOD, it has good results to assay erythrocytes, platelets and blood serum (or plasma) of various animals; homogenate of several dozens types of tissues such as cardiac muscle, lung, liver, kidney, spleen, eardrum, adrenal gland; mitochondrions, microsomes, isolated cardiac muscle perfusate, kidney dialysate, hydroperitoneum, hydrothorax, cerebrospinal fluid, cultured cardiac muscle cells, cultured tumor cells, etc.
9. **Low cost:** Key raw materials of this kit are imported from USA, but kit's price is similar to domestic pyrogallic acid assay method.
10. **No requirement of expensive or special instruments**: it only needs thermostatic water bath and visible range spectrophotometer. Use simple instruments to measure high-level index.

APPENDIX: Experimental Methodology

Content includes erythrocytes SOD extraction, extraction of leucocytes and platelets, homogenate preparation, extraction of mitochondrions and microsomes, microscale protein assay. If you require reference materials such as preparation of erythrocyte membrane and cardiac muscle cell membrane, introduction and relations of free radicals, then you can contact us by telephone or E-mail. We go with your scientific research work certainly, welcome to use our kits.

Customer who require reagents can despatch messenger or use telephone or E-mail to order goods, we can send reagents by mail or delivery to your door.

This method is identified by experts in health department of Jiangsu province, it also get first grade prize for technical achievements of health department.

This international patent is already collected by national patent journal in China, USA, UK, etc.

Address:Central Road, Xuanwu District, Nanjing Xinliji House 258-27 Postal code:210009 Fax:025-83227943
E-Mail:njjcbio@vip.163.com Tel:025-83360321、83360969、83551389 Technology:025-83360272

APPENDIX I: SOD Standard Curve Preparation

1. Pretreatment:

1μg/ml standard solution preparation: Transfer 1mg SOD standard powder produced by a German company named BOEHRINGER MANNHEIM（activity is 5000U/mg）to a volumetric flask, add distilled water until 10ml to prepare 100μg/ml standard stock solution. Transfer 1ml standard stock solution to a volumetric flask, add double distilled water until 100ml to prepare 1μg/ml standard working solution (5U/ml).

2. Operation table:

Tube No.	0	1	2	3	4	5	6	7	8	9	10
Reagent 1 working solution (ml)	1.0	1.0	1.0	1.0	1.0	1.0	1.0	1.0	1.0	1.0	1.0
1μg/ml standard (ml)	0	0.0025	0.005	0.01	0.02	0.03	0.04	0.05	0.06	0.07	0.08
Double distilled water (ml)	0.08	0.0775	0.075	0.07	0.06	0.05	0.04	0.03	0.02	0.01	0.0
Reagent 2 (ml)	0.1	0.1	0.1	0.1	0.1	0.1	0.1	0.1	0.1	0.1	0.1
Reagent 3 (ml)	0.1	0.1	0.1	0.1	0.1	0.1	0.1	0.1	0.1	0.1	0.1
Reagent 4 (ml)	0.1	0.1	0.1	0.1	0.1	0.1	0.1	0.1	0.1	0.1	0.1
Mix sufficiently by vortex, place in 37℃ water bath for 40 minutes											
Chromogenic agent （ml）	2.0	2.0	2.0	2.0	2.0	2.0	2.0	2.0	2.0	2.0	2.0
Mix sufficiently, transfer in cuvettes of 1cm light path, measure absorbances at 550nm (adjust zero by double distilled water)											

3. Result:

Tube No.	0	1	2	3	4	5	6	7	8	9	10
Absorbances	0.550	0.520	0.488	0.437	0.334	0.251	0.173	0.127	0.103	0.097	0.094
Inhibition ratio (%)	0	5.45	11.27	20.55	39.27	54.36	68.55	76.91	81.27	82.36	82.91
Corresponding U	0	0.0125	0.025	0.05	0.10	0.15	0.20	0.25	0.30	0.35	0.40
Corresponding U / ml	0	0.15625	0.3125	0.625	1.25	1.875	2.5	3.125	3.75	4.375	5.0

Address:Central Road, Xuanwu District, Nanjing Xinliji House 258-27 Postal code:210009 Fax:025-83227943
E-Mail:njjcbio@vip.163.com Tel:025-83360321、83360969、83551389 Technology:025-83360272

4. Draw standard curve:

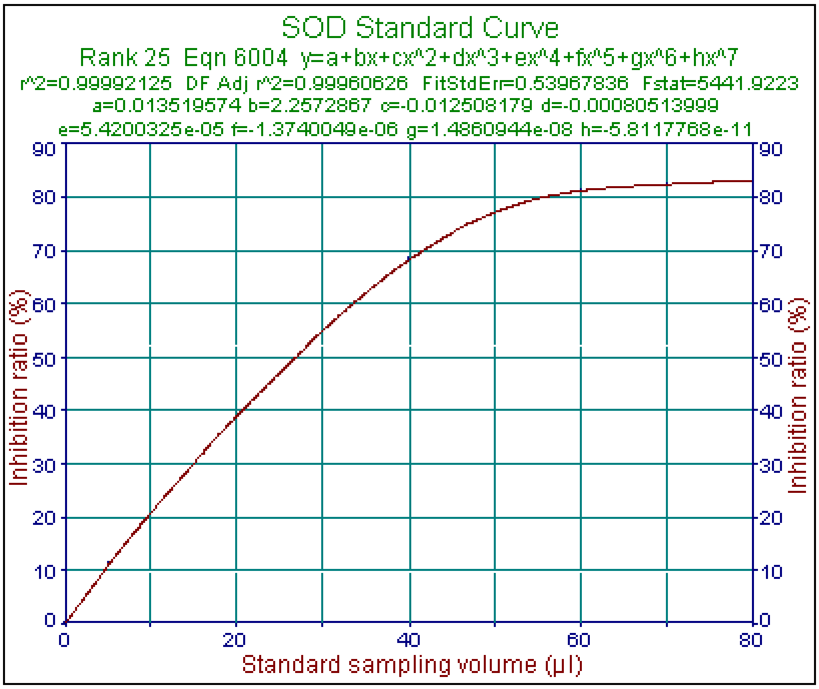

Address:Central Road, Xuanwu District, Nanjing Xinliji House 258-27 Postal code:210009 Fax:025-83227943
E-Mail:njjcbio@vip.163.com Tel:025-83360321、83360969、83551389 Technology:025-83360272

APPENDIX II: T-SOD Assay in Blood Serum (or plasma) and other Transparent Liquids

1. Sample pretreatment:

Pretreatments of cardiac muscle perfusate, kidney dialysate, cell culture fluid should according to blood serum (or plasma).

Take blood serum(or plasma) directly, do optimal sample volume probing according to **Page 6 & 7**. After determine optimal sample volume, you can start formal experiment according to table below.

Operation table:

Reagent	Sample tube	Contrast tube
Reagent 1 working solution (ml)	1.0	1.0
Liquid sample (ml)	a*	
Double distilled water (ml)		a*
Reagent 2 (ml)	0.1	0.1
Reagent 3 (ml)	0.1	0.1
Reagent 4 (ml)	0.1	0.1
Mix sufficiently by vortex, place in 37℃ thermostatic water bath or air bath for 40 minutes		
Chromogenic agent(ml)	2	2
Mix sufficiently, place at room temperation for 10 minutes, transfer to cuvettes of 1cm light path, measure absorbances at 550nm (adjust zero by distilled water).		

3. Calculation:

Definition: Corresponding quantity of SOD that its inhibition ratio pecentage reaches 50% per ml reaction solution is considered as one SOD activity unit (U).

Formula of T-SOD activity in blood serum (or plasma), cardiac muscle perfusate, kidney dialysate, cell culture fluid, etc.:

$$\text{T-SOD activity (U/ml)} = \frac{A_{\text{Contrast}} - A_{\text{Sample}}}{A_{\text{Contrast}}} \div 50\% \times \text{Reaction system dilution times} \times \text{Sample dilution times before assay}$$

4. Examples:

① Take 30μl human blood serum to measure T-SOD activity. In results, $A_{Contrast}$ is 0.476, A_{Sample} is 0.281. Calculate as follows:

Address:Central Road, Xuanwu District, Nanjing Xinliji House 258-27 Postal code:210009 Fax:025-83227943
E-Mail:njjcbio@vip.163.com Tel:025-83360321、83360969、83551389 Technology:025-83360272

$$\text{T-SOD activity (U/ml)} = \frac{0.476 - 0.281}{0.476} \div 50\% \times \frac{3.33\ \text{(Reaction system volume)}}{0.03\ \text{(Sampling volume)}}$$

$$= 90.95\ \text{U/ml}$$

② Take 100μl cell culture fluid to measure T-SOD activity. In results, $A_{Contrast}$ is 0.473, A_{Sample} is 0.312.

Calculate as follows:

$$\text{T-SOD activity (U/ml)} = \frac{0.473 - 0.312}{0.473} \div 50\% \times \frac{3.4\ \text{(Reaction system volume)}}{0.1\ \text{(Sampling volume)}}$$

$$= 23.146\ \text{U/ml}$$

Address:Central Road, Xuanwu District, Nanjing Xinliji House 258-27 Postal code:210009 Fax:025-83227943
E-Mail:njjcbio@vip.163.com Tel:025-83360321、83360969、83551389 Technology:025-83360272

APPENDIX III: Animal tissue T-SOD Assay

1. Sample pretreatment:

Make tissue homogenate according to **Experimental Methodology** (add-on with our goods): Weigh tissue accurately, mix tissue sample and homogenate medium at mass-volume ratio of 1:9 (1g tissue vs 9ml homogenate medium, etc. it is suggested to use 0.86% or 0.9% physiological saline as homogenate medium), use mechanical homogenizer to make 10% homogenate in ice water bath. Centrifugate at 2500~3000rpm for 10 minutes, take supernatant for assay.

Use homogenate medium to dilute supernatant to different concentrations, do optimal sampe volume probing according to **Page 6 & 7**. After determine optimal sample volume, you can start formal experiment according to table below.

2. Operation table:

Reagent	Sample tube	Contrast tube
Reagent 1 working solution (ml)	1.0	1.0
Liquid sample (ml)	a*	
Double distilled water (ml)		a*
Reagent 2 (ml)	0.1	0.1
Reagent 3 (ml)	0.1	0.1
Reagent 4 (ml)	0.1	0.1
Mix sufficiently by vortex, place in 37℃ thermostatic water bath or air bath for 40 minutes		
Chromogenic agent (ml)	2	2
Mix sufficiently, place at room temperation for 10 minutes, transfer to cuvettes of 1cm light path, measure absorbances at 550nm (adjust zero by distilled water).		

3. Calculation:

Definition: Corresponding quantity of SOD that its inhibition ratio pecentage reaches to 50% per mg tissue protein in 1 ml reaction solution is considered as one SOD activity unit (U).

Calculating formula of T-SOD activity in tissue:

$$\text{T-SOD activity (U/ml)} = \frac{A_{Contrast} - A_{Sample}}{A_{Contrast}} \div 50\% \times \frac{\text{Reaction system volume (ml)}}{\text{Sampling volume (ml)}} \div \text{Protein concentration in sample to assay (mgprot / ml)}$$

* mgprot means protein in milligrams.

Address:Central Road, Xuanwu District, Nanjing Xinliji House 258-27 Postal code:210009 Fax:025-83227943
E-Mail:njjcbio@vip.163.com Tel:025-83360321、83360969、83551389 Technology:025-83360272

4. Examples:

① Take 10μl 1% liver tissue homogenate to measure T-SOD activity. In results, $A_{Contrast}$ is 0.510, A_{Sample} is 0.242, protein concentration is 1.075mg/ml. Calculate as follows:

$$\text{T-SOD activity (U/ml)} = \frac{A_{Contrast} - A_{Sample}}{A_{Contrast}} \div 50\% \times \frac{\text{Reaction system volume (ml)}}{\text{Sampling volume (ml)}} \div \text{Protein concentration in sample to assay (mgprot / ml)}$$

$$= \frac{0.510 - 0.242}{0.510} \div 50\% \times \frac{3.31}{0.01} \div 1.075$$

$$= 323.60 \text{ U / mgprot}$$

② Take 50μl 10% earthworm homogenate to measure T-SOD activity. In results, $A_{Contrast}$ is 0.546, A_{Sample} is 0.320, protein concentration in 10% earthworm homogenate is 5.932mg/ml. Calculate as follows:

$$\text{T-SOD activity (U/ml)} = \frac{A_{Contrast} - A_{Sample}}{A_{Contrast}} \div 50\% \times \frac{\text{Reaction system volume (ml)}}{\text{Sampling volume (ml)}} \div \text{Protein concentration in sample to assay (mgprot / ml)}$$

$$= \frac{0.546 - 0.320}{0.546} \div 50\% \times \frac{3.35}{0.05} \div 5.932$$

$$= 9.350 \text{ U / mgprot}$$

③ Take 50μl 5% rabbit retina homogenate to measure T-SOD activity. In results, $A_{Contrast}$ is 0.473, A_{Sample} is 0.271, protein concentration in 5% rabbit retina homogenate is 0.3378 mg/ml. Calculate as follows:

$$\text{T-SOD activity (U/ml)} = \frac{A_{Contrast} - A_{Sample}}{A_{Contrast}} \div 50\% \times \frac{\text{Reaction system volume (ml)}}{\text{Sampling volume (ml)}} \div \text{Protein concentration in sample to assay (mgprot / ml)}$$

$$= \frac{0.473 - 0.271}{0.473} \div 50\% \times \frac{3.35}{0.05} \div 0.3378$$

$$= 169.408 \text{ U/mgprot}$$

④ Take 50μl 0.5% *Acipenser sinensis* liver tissue homogenate to measure T-SOD activity. In results, $A_{Contrast}$ is 0.506, A_{Sample} is 0.326, protein concentration in 0.5% *Acipenser sinensis* liver tissue homogenate is 0.4512 mg/ml. Calculate as follows:

Address:Central Road, Xuanwu District, Nanjing Xinliji House 258-27 Postal code:210009 Fax:025-83227943
E-Mail:njjcbio@vip.163.com Tel:025-83360321、83360969、83551389 Technology:025-83360272

$$\text{T-SOD activity (U/ml)} = \frac{A_{Contrast} - A_{Sample}}{A_{Contrast}} \div 50\% \times \frac{\text{Reaction system volume (ml)}}{\text{Sampling volume (ml)}} \div \text{Protein concentration in sample to assay (mgprot / ml)}$$

$$= \frac{0.506 - 0.326}{0.506} \div 50\% \times \frac{3.35}{0.05} \div 0.4512$$

$$= 105.647 \text{ U/mgprot}$$

⑤ Collect a certain amount of fish roe, add 500μl physiological saline accurately, make homogenate and centrifugate at 2500rpm for 10 minutes. Take supernatant, dilute 5 times with physiological saline, take 50μl to measure T-SOD activity. In results, $A_{Contrast}$ is 0.561, A_{Sample} is 0.309, protein concentration in homogenate supernatant is 3.1062 mg/ml. Calculate as follows:

$$\text{T-SOD activity (U/ml)} = \frac{A_{Contrast} - A_{Sample}}{A_{Contrast}} \div 50\% \times \frac{\text{Reaction system volume (ml)}}{\text{Sampling volume (ml)}} \div \text{Protein concentration in sample to assay (mgprot / ml)}$$

$$= \frac{0.561 - 0.309}{0.561} \div 50\% \times \frac{3.35}{0.05} \div (3.1062 \div 5)$$

$$= 96.891 \text{ U/mgprot}$$

Address:Central Road, Xuanwu District, Nanjing Xinliji House 258-27 Postal code:210009 Fax:025-83227943
E-Mail:njjcbio@vip.163.com Tel:025-83360321、83360969、83551389 Technology:025-83360272

APPENDIX IV: T-SOD assay in hyperlipidemia blood serum (or plasma)

1. Pretreatments of hyperlipidemia blood serum (or plasma):

Slight hyperlipidemia: There is slight turbidity in appearance, it only needs to dilute serum with physiological saline of equivalent volume before assay;

Midrange hyperlipidemia: This is obviously turbidity in appearance, in pretreatment, it needs to add physiological saline of equivalent volume to serum, then add dehydrated ethanol of half volume (volume ratio) of serum, mix well, then you can start assay;

Heavy hyperlipidemia: Take 50μl hyperlipidemia blood serum (or plasma), add 200μl physiological saline, mix sufficiently, add 150μl dehydrated ethanol, mix sufficiently for 1 minute, add 150μl trichloromethane, mix sufficiently for 1 minute, centrifugate at 3500rpm (desk centrifuge) for 10 minutes, take supernatant to assay.

Do optimal sample volume probing according to **Page 6 & 7**. After determine optimal sample volume, you can start formal experiment according to table below.

2. Operation method:

Hyperlipidemia blood serum (or plasma) T-SOD activity assay:

Reagents	Assay tube	Contrast tube
Reagent 1 working solution（ml）	1.0	1.0
Sample（ml）	a*	
Reagent 2（ml）	0.1	0.1
Reagent 3（ml）	0.1	0.1
Reagent 4 working solution（ml）	0.1	0.1

Mix sufficiently by vortex, place in 37℃ thermostatic water bath for 40 minutes.

Chromogenic agent（μl）	2	2
Sample (ml)		a*
Mix sufficiently, place at room temperation for 10 minutes, transfer to cuvettes of 1cm light path, measure absorbances at 550nm (adjust zero by double distilled water).		

3. Calculation of hyperlipidemia blood serum (or plasma) SOD activity:

Definition: Corresponding quantity of SOD that its inhibition ratio pecentage reaches to 50% per ml reaction solution is considered as one SOD activity unit (U).

Address:Central Road, Xuanwu District, Nanjing Xinliji House 258-27 Postal code:210009 Fax:025-83227943
E-Mail:njjcbio@vip.163.com Tel:025-83360321、83360969、83551389 Technology:025-83360272

Formulas of hyperlipidemia blood serum (or plasma) SOD activity:

$$\text{SOD activity (U/ml)} = \frac{A_{Contrast} - A_{Sample}}{A_{Contrast}} \div 50\% \times \text{Reaction system* dilution times} \times \text{Sample dilution times before assay**}$$

$$\text{* Reaction system dilution times} = \frac{\text{Reaction solution volume}}{\text{Sample volume}} = \frac{\text{Reagent volume (3.3ml) + Sample volume}}{\text{Sample volume}}$$

$$\text{** Heavy hyperlipidemia serum (or plasma) dilution times} = \frac{\text{Sample volume (50μl) + Physiological saline (200μl) + Dehydrated alcohol (150μl)}}{\text{Sample volume}} = \frac{400\mu l}{50\mu l}$$

4. Take 50µl rabbit hyperlipidemia blood serum to measure T-SOD activity add 200µl physiological saline, mix sufficiently, add 150µl dehydrate alcohol, mix sufficiently for 1 minute, add 150µl trichloromethane, mix sufficiently for 1 minute, centrifugate at 3500rpm for 10 minutes, take 150 µl to measure. In results, $A_{Contrast}$ is 0.434, A_{Sample} is 0.262. Calculate as follows:

$$\text{T-SOD activity (U/ml)} = \frac{A_{Contrast} - A_{Sample}}{A_{Contrast}} \div 50\% \times \text{Reaction system dilution times} \times \text{Sample dilution times before assay}$$

$$= \frac{0.434 - 0.262}{0.434} \div 50\% \times \frac{\text{3.45 (Reaction solution volume)}}{\text{0.15(Sample volume)}} \times \frac{400\mu l}{50\mu l}$$

$$= 145.84 \text{ U/ml}$$

Address:Central Road, Xuanwu District, Nanjing Xinliji House 258-27 Postal code:210009 Fax:025-83227943
E-Mail:njjcbio@vip.163.com Tel:025-83360321、83360969、83551389 Technology:025-83360272

APPENDIX V: Erythrocytes SOD assay

1. Pretreatments of sample (erythrocytes extract preparation):

(1) Collect 50μl fresh blood or venous blood/ arterial blood anticoagulated by heparin, transfer in 1~2ml physiological saline in centrifuge tube with scales, centrifugate at 500~1000rpm for 10 minutes.

(2) Use micropipette or syringe with anesthetic needle to remove supernatant (must remove totally), keep sediment of erythrocytes.

(3) Add 0.2ml cold double distilled water to sediment of erythrocytes, mix well (let erythrocytes dissolve sufficiently: watch hemolysate towards light, if it becomes transparent, then it can be considered as already dissolved sufficiently).

(4) Add 0.1ml 95% ethanol, shake for 30 seconds.

(5) Add 0.1ml trichloromethane (chloroform), mix sufficiently by vortex for 1 minute.

(6) Centrifugate at 3500rpm for 8 minutes, at this time liquid becomes 3 layers: upper layer is SOD extract, middle layer is hemoglobin sediment, underlayer is trichloromethane. If there is turbidity in supernatant, then you can add a small quantity of solid NaCl and centrifuge for 5 minutes, supernatant will become limpid. Record volume of supernatant, take 5~20μl. **Do optimal sample volume probing according to Page 6 & 7. After determine optimal sample volume, you can start formal experiment according to table below (Residuary erythrocyte extract can be frozen in fridge for next assay).**

[Note] You can get endocanthion blood from rats and mice, use capillary glass tube to impale from endocanthion towards throat, or collect blood by cutting tail, drop blood on adust glass slide with heparin (adust temperature should be lower than 80℃), take 50μl blood by transferpettor to SOD extract. If blood volume is small, then take 10~20μl anticoagulated whole blood. (In chronic animal experiments, you can take blood repeatedly (5~6 times) during feeding period).

Address:Central Road, Xuanwu District, Nanjing Xinliji House 258-27 Postal code:210009 Fax:025-83227943
E-Mail:njjcbio@vip.163.com Tel:025-83360321、83360969、83551389 Technology:025-83360272

2. Operation method: erythrocytes SOD assay:

Reagents	Sample tube	Contrast tube
Reagent 1 (ml)	1.0	1.0
Sample (ml)	a*	
Double distilled water (ml)		a*
Reagent 2 (ml)	0.1	0.1
Reagent 3 (ml)	0.1	0.1
Reagent 4 (ml)	0.1	0.1
**Mix sufficiently by vortex, place in 37℃ for 40 minutes**		
Chromogenic agent(ml)	2	2
Mix sufficiently, place at room temperation for 10 minutes, transfer to cuvettes of 1cm light path, measure absorbances at 550nm (adjust zero by double distilled water).		

3. Calculation of erythrocytes SOD activity:

Definition:Corresponding quantity of SOD that its inhibition ratio pecentage reaches to 50% per g hemoglobin in 1ml reaction solution is considered as one SOD activity unit (U).

Formula:

$$\text{Erythrocyte SOD activity (U/ gHb)} = \frac{A_{Contrast} - A_{Sample}}{A_{Contrast}} \div 50\% \times \text{Reaction solution volume} \times \frac{\text{Total volume of extract}}{\text{Volume of extract to assay}} \times \frac{\text{1ml}}{\text{Blood sample volume}} \div \text{Hemoglobin content (gHb/ml)}$$

4. Examples:

Take 10μl erythrocyte extract to measure T-SOD activity. In results, $A_{Contrast}$ is 0.465, A_{Sample} is 0.293, hemoglobin content in whole blood is 105g/L (0.105g/ml). Calculate as follows:

$$\text{T-SOD activity in erythrocytes (U/gHb)} = \frac{0.465-0.293}{0.465} \div 50\% \times 3.31 \times \frac{0.3^{**}}{0.01^{***}} \times \frac{1}{0.05^{*}} \div 0.105$$

$$=1469.21\ \text{U/ml} \div 0.105\text{gHb/ml} = 13992.50\ \text{U/gHb}$$

* Blood collection volume is 50μl;

** Use 50μl whole blood to do SOD erythrocyte extraction, 300μl extract is made;

*** volume of erythrocyte extract to assay is 10μl .

APPENDIX VI: Plant tissue T-SOD Assay

1. Sample pretreatment:

Make tissue homogenate according to **Experimental Methodology** (add-on with our goods): Weigh tissue accurately, mix tissue sample and homogenate medium at mass-volume ratio of 1:4 (1g tissue vs 4ml homogenate medium, etc. it is suggested to use 0.1mol/L phosphate buffer pH7.0~7.4 as homogenate medium), use mechanical homogenizer to make 20% homogenate in ice water bath. Centrifugate at 3500~4000rpm for 10 minutes, take supernatant for assay. Some medical material and plant powder can be treated by same method.

Use homogenate medium to dilute supernatant to different concentrations, do optimal sampe volume probing according to **Page 6 & 7**. After determine optimal sample volume, you can start formal experiment according to table below.

2. Operation table:

Reagent	Sample tube	Contrast tube
Reagent 1 working solution(ml)	1.0	1.0
Liquid sample (ml)	a*	
Double distilled water (ml)		a*
Reagent 2 (ml)	0.1	0.1
Reagent 3 (ml)	0.1	0.1
Reagent 4 (ml)	0.1	0.1
Mix sufficiently by vortex, place in 37℃ thermostatic water bath or air bath for 40 minutes		
Chromogenic agent (ml)	2	2
Mix sufficiently, place at room temperation for 10 minutes, transfer to cuvettes of 1cm light path, measure absorbances at 550nm (adjust zero by distilled water).		

3. Calculation:

Method 1: Calculate according to tissue wet weight

Definition: Corresponding quantity of SOD that its inhibition ratio pecentage reaches to 50% per g tissue in 1ml reaction solution is considered as one SOD activity unit (U).

Formula:

$$\text{T-SOD activity (U/g wet tissue)} = \frac{A_{\text{Contrast}} - A_{\text{Sample}}}{A_{\text{Contrast}}} \div 50\% \times \frac{\text{Total volume of reaction solution(ml)}}{\text{Sample volume (ml)}} \div \text{Homogenate concentration (g/ml)}$$

Address:Central Road, Xuanwu District, Nanjing Xinliji House 258-27 Postal code:210009 Fax:025-83227943
E-Mail:njjcbio@vip.163.com Tel:025-83360321、83360969、83551389 Technology:025-83360272

Note:

$$\text{Homogenate concentration (g/ml)} = \frac{\text{Tissue wet weight (g)}}{\text{Homogenate volume (ml)}}$$

Method 2: Calculate according to protein concentration

Definition: Corresponding quantity of SOD that its inhibition ratio pecentage reaches to 50% per mg tissue protein in 1ml reaction solution is considered as one SOD activity unit (U).

Formula:

$$\text{T-SOD activity (U/mgprot)} = \frac{A_{Contrast} - A_{Sample}}{A_{Contrast}} \div 50\% \times \frac{\text{Total volume of reaction solution(ml)}}{\text{Sample volume (ml)}} \div \text{Tissue protein concentration (mgprot/ml)}$$

* mgprot means milligram protein

2. Examples

① **Take 50μl** *Arabidopsis thaliana* leave homogenate supernatant to measure T-SOD activity, in results, AContrast is 0.540, ASample is 0.274, tissue protein concentration is 1.3428mg/ml, calculate as follows:

Use method 1:

$$\text{T-SOD activity (U/g wet tissue)} = \frac{A_{Contrast} - A_{Sample}}{A_{Contrast}} \div 50\% \times \frac{\text{Total volume of reaction solution(ml)}}{\text{Sample volume (ml)}} \div \text{Homogenate concentration (g/ml)}$$

$$= \frac{0.540 - 0.274}{0.540} \div 50\% \times \frac{3.35}{0.05} \div \frac{0.1g}{0.9ml}$$

$$= 594.07\ \text{U/g wet tissue}$$

Use method 2:

$$\text{T-SOD activity (U/mgprot)} = \frac{A_{Contrast} - A_{Sample}}{A_{Contrast}} \div 50\% \times \frac{\text{Total volume of reaction solution(ml)}}{\text{Sample volume (ml)}} \div \text{Tissue protein concentration (mgprot/ml)}$$

$$= \frac{0.540 - 0.274}{0.540} \div 50\% \times \frac{3.35}{0.05} \div 1.3428$$

$$= 49.16\ U/mgprot$$

② Take 50μl 5% rice leave homogenate to measure T-SOD, in results, $A_{Contrast}$ is 0.555, A_{Sample} is 0.273, homogenate protein concentration is 0.9833mg/ml, calculate as follows:

Address:Central Road, Xuanwu District, Nanjing Xinliji House 258-27 Postal code:210009 Fax:025-83227943
E-Mail:njjcbio@vip.163.com Tel:025-83360321、83360969、83551389 Technology:025-83360272

Use method 1:

$$\text{T-SOD activity (U/g wet tissue)} = \frac{A_{Contrast} - A_{Sample}}{A_{Contrast}} \div 50\% \times \frac{\text{Total volume of reaction solution(ml)}}{\text{Sample volume (ml)}} \div \text{Homogenate concentration (g/ml)}$$

$$= \frac{0.555-0.273}{0.555} \div 50\% \times \frac{3.35}{0.05} \div \frac{0.1g}{1.9ml}$$

$$= 1293.64 \text{U/g wet tissue}$$

Use method 2:

$$\text{T-SOD activity (U/mgprot)} = \frac{A_{Contrast} - A_{Sample}}{A_{Contrast}} \div 50\% \times \frac{\text{Total volume of reaction solution(ml)}}{\text{Sample volume (ml)}} \div \text{Tissue protein concentration (mgprot/ml)}$$

$$= \frac{0.555-0.273}{0.555} \div 50\% \times \frac{3.35}{0.05} \div 0.9833$$

$$= 69.24\ U/mgprot$$

③ Weigh 0.05g extract powder accurately, add homogenate medium until volume reaches 10ml to dissolve. Take 50μl solution to measure T-SOD activity, in results, $A_{Contrast}$ is 0.537, A_{Sample} is 0.296, calculate as follows:

$$\text{T-SOD activity (U/g)} = \frac{A_{Contrast} - A_{Sample}}{A_{Contrast}} \div 50\% \times \text{Reaction system dilution times} \times \text{Homogenate concentration (g/ml)}$$

$$= \frac{0.537-0.296}{0.537} \div 50\% \times \frac{3.35}{0.05} \div \frac{0.05g}{10ml}$$

$$= 12027.56\ U/g$$

Address:Central Road, Xuanwu District, Nanjing Xinliji House 258-27 Postal code:210009 Fax:025-83227943
E-Mail:njjcbio@vip.163.com Tel:025-83360321、83360969、83551389 Technology:025-83360272
