# Supplementary material for: Dietary Klebsormidium sp. Supplementation Improves Growth Performance, Antioxidant and Anti-Inflammatory Status, Metabolism, and Mid-Intestine Morphology of Litopenaeus Vannamei
Source: Front Nutr. 2022 May 12;9:857351. doi: 10.3389/fnut.2022.857351 (PMC9136981; doi:10.3389/fnut.2022.857351)
Supplement: Supplementary file 3 [file Data_Sheet_3.pdf]

Glutathione Peroxidase（GSH-PX）Assay Kit

(Colorimetric Method)

Serial No: A005 Pack：50T/24S 100T/48S

Catalogue

Assay significance ········· 2
Assay principle ········· 2
Kit expiry date and storage condition ········· 2
Pre-test announcements ········· 2
Reagent composition and preparation ········· 3
Whole blood GSH-PX activity assay ········· 6
GSH-PX activity assay in tissues, mitochondria and cell membranes ········· 9
Blood serum (or plasma) GSH-PX activity assay ········· 14
Announcement ········· 17
Appendix: GSH standard curve preparation ········· 18

Address:Central Road, Xuanwu District, Nanjing Xinliji House 258-27 Postal code:210009 Fax:025-83227943
E-Mail:njjcbio@vip.163.com Tel:025-83360321、83360969、83551389 Technology:025-83360272

Assay significance

Glutathione peroxidase (GSH-PX) is a widespread, important hydrogen peroxidase in organisms. It catalyzes reduced glutathione’s reduction reaction with peroxide diagnostically leads to protection of cell structure and functions. GSH-PX’s active site is Se-cysteine, selenium is necessary part of GSH-PX, 1 mol GSH-PX contains 4mol selenium. GSH-PX activity can be used as a biochemical indicator of Se level in vivo.

Assay principle

Glutathione peroxidase (GSH-PX) can catalyze peroxide (H2O2) and reduced glutathione (GSH) to produce H_2O and oxidized glutathione (GSSG). GSH-PX activity can be represented by its enzymatic reaction rate. It is able to calculate enzyme activity by measuring GSH consumption in this enzymatic reaction.

GSH-PX activity is represented by catalyzed GSH reaction rate. These two substrates can undertake

$$H_2O_2 + 2GSH \xrightarrow{GSH-PX} 2H_2O + GSSG$$

redox reaction without enzyme (non-enzymatic reaction), so GSH consumption caused by non-enzymatic reaction must be deducted when you calculate enzyme activity in conclusion.

GSH content assay: GSH reacts with dithio-dinitrobenzoic acid to produce 5-thio-dinitrobenzoic acid anions with relatively stable yellow color. It is able to calculate GSH content by measuring absorbance at 412nm.

Kit expiry date and storage condition

The whole kit can be stored at 4℃ for 6 months.

Address:Central Road, Xuanwu District, Nanjing Xinliji House 258-27 Postal code:210009 Fax:025-83227943
E-Mail:njjcbio@vip.163.com Tel:025-83360321、83360969、83551389 Technology:025-83360272

Pre-test announcements

Note1: You only need to make 1~2 blank tubes and standard tubes for each batch.

Note 2: Different samples have different GSH-PX activities, so they also have different optimal sample concentrations. According to parabola proportion between inhibition percentage and enzyme activity, different samples have different sample volumes and different sample concentration, you need to determine an optimal sample volume and an optimal sample concentration before measure GPX activity in a new sample.

Optimal sample probing:

Do pre-test to determine optimal sample concentration: If you use this kit to do a new sample assay, then you need to make 3 sample tubes of different concentrations to do pre-test as follows:

Whole blood: Take 200μl 1:24, 1:49, 1:99 hemolysate (diluted by double distilled water) separately to do pre-test.

Tissue homogenate: Take 200μl 10%, 5%, 1% homolysate (different samples need different dilutions) separately to do pre-test.

Blood serum: Take 100μl undiluted, 1:1, 1:4, 1:9, 1:19 blood serum (diluted by physiological saline) to do pre-test.

Then calculate: Inhibition percentage=(ODContrast-ODSample)/ODContrast X 100%, result should be in range of **15%~55%**. Take the tube which inhibition percentage is between 45% to 50% as **optimal sample volume.**

Enzyme inhibition percentage and enzyme activity appear parabola proportion. If inhibition percentage is over 60% (curve appears flat in this part), then you need to dilute sample or decrease sample volume and try again. If inhibition percentage is lower than 20%, then you need to increase sample volume and try again.

In this way, it is great helpful for scientific result analysis and t-test; if inhibition ratio percentage is higher than 60% or lower than 10%, then there are no significant differences between different assay groups.

Address:Central Road, Xuanwu District, Nanjing Xinliji House 258-27 Postal code:210009 Fax:025-83227943
E-Mail:njjcbio@vip.163.com Tel:025-83360321、83360969、83551389 Technology:025-83360272

Reagent composition and preparation

100T/48S:

	Reagent No.	Name	Quantity and storage condition
1	**Reagent 1**	Stock solution	2ml×1 bottle, can be stored at 4℃ for 6 months
	**Reagent 1 working solution preparation:** Take 0.1ml stock solution, add double distilled water until volume reaches 10ml, this operation equals to 100 times dilution. Working solution should be used soon after preparation, consider solution volume according to you need. Can be stored at 4℃		
2	**Reagent 2**	Powder A	1 bottle, can be stored at 4℃ for 6 months
		**Powder A:** Add 170 ml hot double distilled water (90~100℃) to dissolve completely.	
		Solution B	50ml×1; can be stored at 4℃ for 6 months.
	**Reagent 2 working solution preparation:** Mix prepared Solution A and Solution B sufficiently. This mixtured is supersaturated solution, so if there are crystals seed out after cooling and placing, then take supernatant for experiment. Can be stored at 4℃ or room temperature for 6 months.		
3	**Reagent 3**	Powder	1 bottle, can be stored at 4℃ for 6 months.
	**Reagent 3 working solution preparation:** Add 200ml double distilled water to dissolve, can be stored in plastic bottle (given by our Institute) for 6 months.		
4	**Reagent 4**	Powder	1 vial, can be stored at 4℃ away from light for 6 months.
	**Reagent 4 working solution preparation:** Add 50ml double distilled water to dissolve, can be stored at 4℃ away from light for 6 months		
5	**Reagent 5**	Powder	4 vials, can be stored at 4℃ away from light for 6 months.
	**Reagent 5 working solution preparation:** Add 10ml double distilled water each vial to dissolve, can be stored at 4℃ away from light for 5 days.		
6	**Reagent 6**	GSH standard powder	3.07mg×4 vials, can be stored at 4℃ for 6 months.
7	**Reagent 7**	GSH standard solvent stock solution	10ml×1 bottle, can be stored at 4℃ for 6 months.
	**Standard solvent working solution preparation:** Mix stock solution with double distilled water at ratio of 1:9 (equals to 10 times dilution) to make working solution. Please use working solution soon after preparation. Can be stored at 4℃.		
8	**1mmol/LGSH solution preparation:** GSH 's molecular weight is 307. Before assay, add 1 vial (3.07mg) GSH standard powder to GSH standard solvent working solution, add solvent until mixture volume reaches 10ml, this solution is considered as 1mmol/L GSH solution. Please use this solution soon after preparation.		
	**100μmol/LGSH solution preparation:** Mix 2ml 1mmol/LGSH solution and 18ml GSH solution working solution, adjust volume to 20ml. This solution is used to draw standard curve. If you don't want to draw standard curve, then you can skip this step. (See in *Appendix V: GSH standard curve preparation*).		
	**20μmol/L GSH standard solution:** Take 0.2ml 1mmol/L GSH solution, add GSH standard solvent working solution until mixture volume reaches to 10ml.This solution is considered as 20μmol/L GSH standard solution.		

Address:Central Road, Xuanwu District, Nanjing Xinliji House 258-27 Postal code:210009 Fax:025-83227943
E-Mail:njjcbio@vip.163.com Tel:025-83360321、83360969、83551389 Technology:025-83360272

50T/24S:

	Reagent No.	Name	Quantity and storage condition
1	**Reagent 1**	Stock solution	1ml×1 bottle, can be stored at 4℃ for 6 months
	**Reagent 1 working solution preparation:** Take 0.1ml stock solution, add double distilled water until volume reaches 10ml, this operation equals to 100 times dilution. Working solution should be used soon after preparation, consider solution volume according to you need. Can be stored at 4℃		
2	**Reagent 2**	Powder A	1 bottle, can be stored at 4℃ for 6 months
		**Powder A:** Add 85ml hot double distilled water (90~100℃) to dissolve completely.	
		Solution B	25ml×1; can be stored at 4℃ for 6 months.
	**Reagent 2 working solution preparation:** Mix prepared Solution A and Solution B sufficiently. This mixture is supersaturated solution, so if there are crystals seed out after cooling and placing, then take supernatant for experiment. Can be stored at 4℃ or room temperature for 6 months.		
3	**Reagent 3**	Powder	1 bottle, can be stored at 4℃ for 6 months.
	**Reagent 3 working solution preparation:** Add 100ml double distilled water to dissolve, can be stored in plastic bottle (given by our Institute) for 6 months.		
4	**Reagent 4**	Liquid	25ml×1 bottle, can be stored at 4℃ away from light for 6 months.
5	**Reagent 5**	Powder	2 vials, can be stored at 4℃ away from light for 6 months.
	**Reagent 5 working solution preparation:** Add 10ml double distilled water each vial to dissolve, can be stored at 4℃ away from light for 5 days.		
6	**Reagent 6**	GSH standard powder	3.07mg×2 vials, can be stored at 4℃ for 6 months.
7	**Reagent 7**	GSH standard solvent stock solution	5ml×1 bottle, can be stored at 4℃ for 6 months.
	**Standard solvent working solution preparation:** Mix stock solution with double distilled water at ratio of 1:9 (equals to 10 times dilution) to make working solution. Please use working solution soon after preparation. Can be stored at 4℃.		
8	**1mmol/LGSH solution preparation:** GSH ‘s molecular weight is 307. Before assay, add 1 vial (3.07mg) GSH standard powder to GSH standard solvent working solution, add solvent until mixture volume reaches 10ml, this solution is considered as 1mmol/L GSH solution. Please use this solution soon after preparation.		
	**100μmol/LGSH solution preparation:** Mix 2ml 1mmol/LGSH solution and 18ml GSH solution working solution, adjust volume to 20ml. This solution is used to draw standard curve. If you don’t want to draw standard curve, then you can skip this step. (See in *Appendix V: GSH standard curve preparation*).		
	**20μmol/L GSH standard solution:** Take 0.2ml 1mmol/L GSH solution, add GSH standard solvent working solution until mixture volume reaches to 10ml.This solution is considered as 20μmol/L GSH standard solution.		

Address:Central Road, Xuanwu District, Nanjing Xinliji House 258-27 Postal code:210009 Fax:025-83227943
E-Mail:njjcbio@vip.163.com Tel:025-83360321、83360969、83551389 Technology:025-83360272

Whole blood GSH-PX activity assay

1. Sample pretreatment:

Hemolysate preparation:

① Take 20μl heparin anticoagulated whole blood, dilute with double distilled water to 1ml, 1:49 hemolysate is made. Dilute 10μl mouse/rat blood with distilled water to 1ml, 1:99 hemolysate is made.

② Mix sufficiently, place for 5 minutes until hemolysate in glass tube appears **completely transparent** towards light, then you can start assay.

③ GSH-PX activity in prepared hemolysate can maintain for only 45~60 minutes (extend to 120 minutes in cold weather. If you have not enough time to assay in the same day, then you can keep you samples in anticoagulated whole blood fridge(4℃～8℃), enzyme activity keeps stable in 2~3 days.

Note 1: Please do preliminary experiment before formal experiment, details in **example of hemolysate optimal sample concentration and optimal sample volume probing.**

Note 2: Please make sure that erythrocytes are completely haemolyzed before measure blood GSH-PX activity (completely haemolyzed blood appears **transparent** towards light, **if it is not transparent enough, then you can freeze-thaw it once**. But erythrocytes of some rats and pigs cannot be placed below 0℃ or they will be hard to haemolyze. For example, erythrocytes of diabetic rats and some normal rats are very hard to haemolyze after freezing. It is better to take 1-2 samples to do pretesting before formal experiment.

2. Operation procedures:

(1) Enzymatic reaction: (Reagent 1 working solution is already prewarmed at 37℃).

	Non-enzyme tube	Enzyme tube
**1mmol/L GSH (ml)**	0.2	0.2
**Hemolysate (ml)**		0.2
Prewarm in 37ºC water bath for 5 minutes.		
**Reagent 1 working solution**	0.1	0.1
React in 37ºC water bath for 5 minutes accurately.		
**Reagent 2 (ml)**	2	2
**Hemolysate (ml)**	0.2	
Mix sufficiently, centrifugate at 3500~4000 rpm for 10 minutes, take 1ml supernatant for chromogenic reaction.		

(2) Chromogenic reaction:

	Blank tube	Standard tube	Non-enzyme tube	Enzyme tube
**GSH standard solvent working solution (ml)**	1			
**20μmol/L GSH standard solution（ml）**		1		
**Supernatant（ml）**			1	1
**Reagent 3 working solution（ml）**	1	1	1	1
**Reagent 4 working solution（ml）**	0.25	0.25	0.25	0.25
**Reagent 5 working solution（ml）**	0.05	0.05	0.05	0.05
Mix sufficiently, place at room temperature for 15 minutes, transfer in cuvettes of 1cm light path, measure OD values of all tubes at 412nm (adjust zero by double distilled water).				

3. Calculation:

Definition: Reacts at 37°C for 5 minutes, 1μmol/L GSH concentration decreasing (already deduct effect of nonenzymatic reaction) in reaction system per 4μl whole blood is considered as 1 enzyme activity unit (U).

Formula:

$$\text{Whole blood GSH-PX activity} = \frac{OD_{\text{Nonenzyme}} - OD_{\text{Enzyme}}}{OD_{\text{Standard}} - OD_{\text{Blank}}} \times \text{Standard solution concentration (20}\mu\text{mol/L)} \times \text{Dilution times } \left(5^{*} \times \frac{1+X^{**}}{1+49^{***}}\right)$$

Note: * According to operation table of enzymatic reaction, 0.5ml reaction solution is mixed with 2ml Reagent 2, this fact equals to 5 times dilution, so multiply with 5.

** X is whole blood dilution times. For example, if you dilute at ratio of 1:99, then X=99.

*** 1+49: When hemolysate is diluted at ratio of 1:49, taking 0.2ml hemolysate to assay equals to taking 4μl whole blood.

4. Example of hemolysate optimal sample concentration and optimal sample volume probing:

(1) Sample source: Fresh normal rat tail whole blood

(2) Sample pretreatment:

Dilute rat whole blood with double distilled water at ratio of 1:24, 1:49, 1:59, 1:69, 1:79, 1:89, 1:99,

Address:Central Road, Xuanwu District, Nanjing Xinliji House 258-27 Postal code:210009 Fax:025-83227943
E-Mail:njjcbio@vip.163.com Tel:025-83360321、83360969、83551389 Technology:025-83360272

1:149, 1:199 separately in order to make a series of hemolysates of different concentrations. Take 0.2ml a series of hemolysates of different concentrations to assay according to whole blood assay operation table.

(3) Operation table:

ODBlank		0.041	
ODStandard		0.163	
Sample concentration	ODEnzyme	ODNon-enzyme	Inhibition percentage
1：24	0.079	0.451	82.48%
1：49	0.096	0.452	78.76%
1：59	0.107	0.450	76.22%
1：69	0.118	0.452	73.89%
1：79	0.130	0.450	71.11%
1：89	0.144	0.450	68.00%
1：99	0.163	0.449	63.70%
**1：149**	**0.231**	**0.450**	**48.67%**
1：199	0.298	0.449	33.63%

(4) Conclusion:

According to data above, optimal sample concentration of 1:149 can make inhibition ratio (inhibition percentage=(($OD_{Contrast}$-OD_{Sample})/$OD_{Contrast}$) ×100%) in the range of 45%～55%. As result, it is better to take 0.2ml 1:149 diluted normal rat tail whole blood for GSH-PX formal experiment.

(5) Example:

Example 1: Take 0.2ml 1:49 diluted hemolysate to assay. In results, $OD_{Nonenzyme}$ is 0.463, OD_{Enzyme} is 0.228, $OD_{Standard}$ is 0.165, OD_{Blank} is 0.041. Calculate as follows:

$$\text{Whole blood GSH-PX activity} = \frac{OD_{Nonenzyme} - OD_{Enzyme}}{OD_{Standard} - OD_{Blank}} \times \text{Standard solution concentration } (20\mu mol/L) \times 5 \times \frac{1+49}{1+49}$$

$$= \frac{0.463-0.228}{0.165-0.041} \times 20 \times 5 \times \frac{50}{50} = 189.52\ U$$

Example 2: Take 0.2ml 1:149 diluted rat hemolysate to assay, length of water bath is 5 minutes. In results, $OD_{Nonenzyme}$ is 0.451, OD_{Enzyme} is 0.205, $OD_{Standard}$ is 0.165, OD_{Blank} is 0.041. Calculate as follows:

$$\text{Whole blood GSH-PX activity} = \frac{OD_{Nonenzyme} - OD_{Enzyme}}{OD_{Standard} - OD_{Blank}} \times \text{Standard solution concentration } (20\mu mol/L) \times 5 \times \frac{1+149}{1+49}$$

$$= \frac{0.451-0.205}{0.165-0.041} \times 20 \times 5 \times \frac{150}{50} = 595.16\ U$$

Address:Central Road, Xuanwu District, Nanjing Xinliji House 258-27 Postal code:210009 Fax:025-83227943
E-Mail:njjcbio@vip.163.com Tel:025-83360321、83360969、83551389 Technology:025-83360272

GSH-PX activity assay in tissues, mitochondria and cell membrane

1. Sample pretreatment:

(1) 10% tissue homogenate preparation:

a. Take tissue piece (0.2~1g), wash by ice-cold physiological saline. Remove blood, dry by filter paper, transfer in 5~10ml small flask.

b. Use cylinder to measure volume of precooled **homogenate medium**(PH7.4，0.01mol/L sucrose，0.01mol/L Tris-HCl，0.0001mol/L EDTA2Na solution) or physiological saline. Volume of homogenate medium or 0.86% physiological saline should be 9 times as tissue weight, transfer 2/3 total volume of homogenate medium or physiological saline into small flask. Cut tissue to small pieces by ophthalmology small scissor as quick as possible (please put small flask in ice water bath in hot weather).

c. Pour small tissue pieces to glass homogenate tube, then wash tissue residual by 1/3 volume of homogenate medium or 0.86% physiological saline, transfer all mixture into glass homogenater tube then start to make homogenate. Use left hand to hold homogenate tube to make sure its bottom in ice water bath, use right hand to insert bar in tube, grind by up-down rolling several dozen times (6~8 minutes), grind sufficient to make sure tissue becomes homogenate. You can also use tissue triturator to grind at 10000～15000r/min to make 10% homogenate; incision tissue homogenizer can also be used (homogenate length: 10 seconds/time, interval is 30 seconds, do 3~4 times at 0～4℃) . Cardiac muscle needs longer homogenate time.

Note: Take optimal concentration to assay (according to pretesting result).

d. Centrifugate prepared 10% homogenate at 3000rpm (by common centrifuge or low temperature centrifuge) for 10~15 minutes. Remove sediment, keep supernatant.

e. Take supernatant for various kinds of assay according to your require.

(2) Mitochondrion preparation:

Take 5~10ml 10% tissue homogenate, centrifugate at 1000~2000rpm for 10 minutes (by common centrifuge or low temperature low speed centrifuge). Take supernatant, centrifugate at 8000~10000rpm (by low temperature high speed centrifuge) for 15 minutes, sediment is mitochondria.

2. Operation procedures:

(1) Enzymatic reaction (Reagent 1 working solution should be prewarmed in 37℃ water bath).

	**Nonenzyme tube**	**Enzyme tube**
**1mmol/L GSH（ml）**	0.2	0.2
**Homogenate （ml）**		0.2
Prewarm in 37ºC water bath for 5 minutes.		
**Reagent 1 working solution（ml）**	0.1	0.1
React in 37ºC water bath for 5 minutes accurately		
**Reagent 2 working solution（ml）**	2	2
**Homogenate （ml）**	0.2	
Mix sufficiently, centrifugate at 3500~4000rpm for 10 minutes, take 1ml supernatant for chromogenic reaction.		

(2) Chromogenic reaction:

	**Blank tube**	**Standard tube**	**Nonenzyme tube**	**Enzyme tube**
**GSH standard solvent working solution**（ml）	1			
**20μmol/L GSH standard solution**（ml）		1		
**Supernatant**（ml）			1	1
**Reagent 3 working solution**（ml）	1	1	1	1
**Reagent 4 working solution**（ml）	0.25	0.25	0.25	0.25
**Reagent 5 working solution**（ml）	0.05	0.05	0.05	0.05
Mix sufficiently, place at room temperature for 15 minutes, transfer in cuvettes of 1cm light path, measure OD values of all tubes at 412nm (adjust zero by double distilled water).				

(3) Calculation:

Definition: 1μmol/L GSH concentration decreasing (already deduct effect of nonenzymatic reaction) in reaction system per mg protein per minute is considered as 1 enzyme activity unit (U).

Note: Tissue protein assay: According to experimental methodology, use biuret method, Coomassie brilliant blue method, ultraviolet method or salicylsulfonic acid method to measure tissue protein content in milligrams.

Formula:

$$\text{Tissue GSH-PX activity} = \frac{OD_{Nonenzyme} - OD_{Enzyme}}{OD_{Standard} - OD_{Blank}} \times \text{Standard solution concentration } (20\mu mol/L) \times \text{Dilution times } (5^*) \div \text{Reaction time length} \div (\text{Sample volume} \times \text{Protein content in sample})$$

Note: * According to operation table of enzymatic reaction, 0.5ml reaction solution is mixed with 2ml Reagent 2, this fact equals to 5 times dilution, so multiply with 5.

(4) Example of tissue homogenate optimal sample concentration and optimal sample volume probing:

① **Sample source:** Use normal mouse liver tissue to make 10% liver homogenat supernatant, dilute with physiological saline to 1% for assay.

② **Sample pretreatment:**

Dilute 1% liver homogenate with physiological saline to 1.0%, 0.5%, 0.4%, 0.3%, 0.25%, 0.1%, 0.05% separately in order to make a series of homogenates of different concentrations. Take 0.2ml a series of homogenates of different concentrations to assay according to tissue assay operation table.

③ **Results:**

ODBlank		0.041	
ODStandard		0.163	
Sample concentration	ODEnzyme	ODNonenzyme	Inhibition percentage
0.05%	0.411	0.463	11.23%
0.1%	0.356	0.464	23.28%
0.2%	0.283	0.462	38.74%
**0.25%**	**0.244**	**0.461**	**47.07%**
0.3%	0.213	0.462	53.89%
0.4%	0.175	0.461	62.04%
0.5%	0.145	0.460	68.47%
1.0%	0.085	0.460	81.52%

④ **Conclusion:**

According to data above, optimal sampling concentration of 0.25%~0.3% can make inhibition ratio (inhibition percentage=(($OD_{Contrast}$-OD_{Sample})/$OD_{Contrast}$) ×100%) in the range of 45%～55%. As result, it is better to take 0.2ml 0.25% normal mouse liver tissue homogenate for GSH-PX formal assay.

Address:Central Road, Xuanwu District, Nanjing Xinliji House158-27 Postal code:210009 Fax:025-83227943
E-Mail:njjcbio@vip.163.com Tel:025-83360321、83360969、83551389 Technology:025-83360272

⑤ **Examples:**

Example 1: Take 0.2ml 0.25% mouse liver tissue homogenate to assay. In results, $OD_{Nonenzyme}$ is 0.480, OD_{Enzyme} is 0.172, $OD_{Standard}$ is 0.163, OD_{Blank} is 0.041, protein content in 1% homogenate is 0.910mg/ml, standard solution concentration is 20μmol/L . Calculate as follows:

$$\text{Liver tissue GSH-PX activity} = \frac{OD_{Nonenzyme} - OD_{Enzyme}}{OD_{Standard} - OD_{Blank}} \times \text{Standard solution concentration } (20\mu mol/L) \times \text{Dilution times}$$

$$\div \text{Reaction time length} \div (\text{Sample volume} \times \text{Protein content in sample})$$

$$= \frac{0.480-0.172}{0.163-0.041} \times 20 \times 5 \div 5 \div (0.910 \div 4^{*} \times 0.2)$$

$$= 1109.71 U$$

Note: * 0.25% mouse liver homogenate is used to measure GSH-PX activity, 1% mouse liver homogenate is used to measure protein content, so in calculation, it should be divided by 4.

Example 2: Take 0.2ml 0.5% crucian liver tissue homogenate to assay. In results, $OD_{Nonenzyme}$ is 0.454, OD_{Enzyme} is 0.264, $OD_{Standard}$ is 0.163, OD_{Blank} is 0.041, protein content in 1% homogenate is 0.8367mg/ml, standard solution concentration is 20μmol/L . Calculate as follows:

$$\text{Liver tissue GSH-PX activity} = \frac{OD_{Nonenzyme} - OD_{Enzyme}}{OD_{Standard} - OD_{Blank}} \times \text{Standard solution concentration } (20\mu mol/L) \times \text{Dilution times}$$

$$\div \text{Reaction time length} \div (\text{Sample volume} \times \text{Protein content in sample})$$

$$= \frac{0.454-0.264}{0.163-0.041} \times 20 \times 5 \div 5 \div (0.8367 \div 2^{*} \times 0.2)$$

$$= 372.266 U$$

Note: * 0.5% crucian liver homogenate is used to measure GSH-PX activity, 1% crucian liver homogenate is used to measure protein content, so in calculation, it should be divided by 2.

Address:Central Road, Xuanwu District, Nanjing Xinliji House 258-27 Postal code:210009 Fax:025-83227943
E-Mail:njjcbio@vip.163.com Tel:025-83360321、83360969、83551389 Technology:025-83360272

Example 3: Take 0.2ml 10% rat testis tissue homogenate to assay. In results, $OD_{Nonenzyme}$ is 0.468, OD_{Enzyme} is 0.258, $OD_{Standard}$ is 0.163, OD_{Blank} is 0.041, protein content in 10% homogenate is 6.941mg/ml, standard solution concentration is 20μmol/L . Calculate as follows:

$$\text{Testis tissue GSH-PX activity} = \frac{OD_{Nonenzyme} - OD_{Enzyme}}{OD_{Standard} - OD_{Blank}} \times \text{Standard solution concentration } (20\mu mol/L) \times \text{Dilution times}$$

$$\div \text{Reaction time length} \div (\text{Sample volume} \times \text{Protein content in sample})$$

$$= \frac{0.468-0.258}{0.163-0.041} \times 20 \times 5 \div 5 \div (6.941 \times 0.2)$$

$$= 24.799\ U$$

Address:Central Road, Xuanwu District, Nanjing Xinliji House 258-27 Postal code:210009 Fax:025-83227943
E-Mail:njjcbio@vip.163.com Tel:025-83360321、83360969、83551389 Technology:025-83360272

Blood serum (or plasma) GSH-PX activity assay

1. Operation procedure:

(1) Enzymatic reaction: (Reagent 1 working solution should be prewarmed at 37ºC water bath)

	Nonenzyme tube	Enzyme tube
**1mmol/L GSH (ml)**	0.2	0.2
**Blood serum (or plasma)(ml)**		0.1
Prewarm in 37ºC water bath for 5 minutes		
**Reagent 1 working solution (ml)**	0.1	0.1
React in 37ºC water bath for 5 minutes accurately.		
**Reagent 2 working solution (ml)**	2	2
**Blood serum (or plasma)(ml)**	0.1	
Mix sufficiently, centrifugate at 3500~4000rpm for 10 minutes, take 1ml supernatant for chromogenic reaction.		

(2) Chromogenic reaction:

	Blank tube	Standard tube	Nonenzyme tube	Enzyme tube
**GSH standard solvent working solution**（ml）	1			
**20μmol/L GSH standard solution**（ml）		1		
**Supernatant**（ml）			1	1
**Reagent 3 working solution**（ml）	1	1	1	1
**Reagent 4 working solution**（ml）	0.25	0.25	0.25	0.25
**Reagent 5 working solution**（ml）	0.05	0.05	0.05	0.05
Mix sufficiently, place at room temperature for 15 minutes, transfer in cuvettes of 1cm light path, measure OD values of all tubes at 412nm (adjust zero by double distilled water).				

2. Calculation:

Definition: Reacts at 37℃ for 5 minutes, 1μmol/L GSH concentration decreasing (already deduct effect of nonenzymatic reaction) in reaction system per 0.1ml blood serum is considered as 1 enzyme activity unit (U).

Address:Central Road, Xuanwu District, Nanjing Xinliji House 258-27 Postal code:210009 Fax:025-83227943
E-Mail:njjcbio@vip.163.com Tel:025-83360321、83360969、83551389 Technology:025-83360272

Formula:

$$\text{Blood serum (or plasma) GSH-PX activity} = \frac{OD_{Nonenzyme} - OD_{Enzyme}}{OD_{Standard} - OD_{Blank}} \times \text{Standard solution concentration } (20\mu mol/L) \times \text{Dilution times } (6^*) \times \text{Sample dilution times before assay}$$

Note: * According to operation table of enzymatic reaction, 0.4ml reaction solution is mixed with 2ml Reagent 2, this fact equals to 6 times dilution, so multiply with 6.

3. Example of blood plasma optimal sample concentration and optimal sample volume probing:

(1) Sample source: Take whole blood from normal rat eye sockets, get blood plasma anticoagulated by heparin.

(2) Sample pretreatment:

Dilute blood plasma with physiological saline at ratio of 1:1, 1:4, 1:7, 1:14, 1:19 separately in order to make a series of blood plasma of different concentrations. Take 0.1ml a series of blood plasma of different concentrations to assay according to blood plasma (serum) assay operation table.

(3) Result:

ODBlank		0.041	
ODStandard		0.163	
Sample concentration	OD_{Enzyme}	$OD_{Nonenzyme}$	Inhibition percentage
1：1	0.117	0.519	77.46%
**1：4**	**0.283**	**0.520**	**45.58%**
1：7	0.365	0.518	29.53%
1：9	0.397	0.518	23.36%
1：14	0.421	0.516	18.41%
1：19	0.442	0.517	14.51%

④ Conclusion:

According to data above, optimal sample concentration of 1:4 can make inhibition percentage (inhibition percentage=((ODContrast-ODSample)/ODContrast) ×100%) in the range of 45%～55%. As result, it is better to take 0.1ml 1:4 diluted normal rat blood plasma for GSH-PX formal assay.

4. Example:

Take 0.1ml 1:4 diluted rat blood serum to assay. In results, ODNonenzyme is 0.475, ODEnzyme is 0.293, ODStandard is 0.165, ODBlank is 0.041. Calculate as follows:

$$\text{Rat blood serum GSH-PX activity} = \frac{0.475-0.293}{0.165-0.041} \times 20 \times 6 \times 5 = 880.65\ U$$

Address:Central Road, Xuanwu District, Nanjing Xinliji House 258-27 Postal code:210009 Fax:025-83227943
E-Mail:njjcbio@vip.163.com Tel:025-83360321、83360969、83551389 Technology:025-83360272

Announcements

(1) Hemolysate enzyme activity keeps stable at room temperature for 1 hour. So it is suggested to measure no longer than 1hour after sample dilution.

(2) Blood samples must be fresh. Heparin anticoagulated blood can be stored at 4～8℃ in fridge less than 48 hours.

(3) Please make sure that erythrocytes are completely haemolyzed before measure blood GSH-PX activity (completely haemolyzed blood appears **transparent** towards light, **if it is not transparent enough, then you can freeze-thaw it once.** But erythrocytes of some rats and swines can not be placed below 0℃ or they will be hard to haemolyze. For example, erythrocytes of diabetic rats and some normal rats are very hard to haemolyze after freezing. It is better to take 1-2 samples to do pretesting before formal experiment.

(4) Reagent 1 container should be washed completely, its working solution should be used soon after preparation.

(5) 1mmol/L GSH, 0.1 mmol/L GSH, 20μmol/L GSH standard solutions should be used soon after preparation.

(6) **Reaction time recording must be accurate** (for example, react in 37℃ water bath for 5 minutes).

(7) Taking and measuring of supernatant should be down in same day.

(8) There are various methods to measure protein content in tissue, you can follow Experimental Methodology or buy our protein assay kits.

Address:Central Road, Xuanwu District, Nanjing Xinliji House258-27 Postal code:210009 Fax:025-83227943
E-Mail:njjcbio@vip.163.com Tel:025-83360321、83360969、83551389 Technology:025-83360272

Appendix : GSH standard curve preparation

1. Operation table:

Take 2ml 1mmol/L GSH standard solution, add 18ml GSH standard solvent to adjust volume to 20ml, 100μmol/L GSH standard working solution is prepared.

Tube No.	1	2	3	4	5	6
**100μmol/L GSH standard（ml）**	0	0.4	0.8	1.2	1.6	2
**Reagent 2 working solution (ml)**	2	1.6	1.2	0.8	0.4	0
**Reagent 3 working solution (ml)**	2	2	2	2	2	2
**Reagent 4 working solution (ml)**	0.5	0.5	0.5	0.5	0.5	0.5
**Reagent 5 working solution (ml)**	0.1	0.1	0.1	0.1	0.1	0.1
Mix sufficiently, place at room temperature for 15 minutes, transfer in cuvettes of 1cm light path, measure OD values of all tubes at 412nm (adjust zero by double distilled water)						

2. Result:

Tube No.	1	2	3	4	5	6
**Corresponding GSH standard concentration（μmol/L）**	0	20	40	60	80	100
**Referenced OD**	0.043	0.165	0.285	0.406	0.525	0.648
**Absolute OD**	0	0.122	0.242	0.363	0.482	0.605

3. Draw graph:

Use corresponding GSH standard concentration as x axis, use absolute OD values as y axis, draw standard curve:

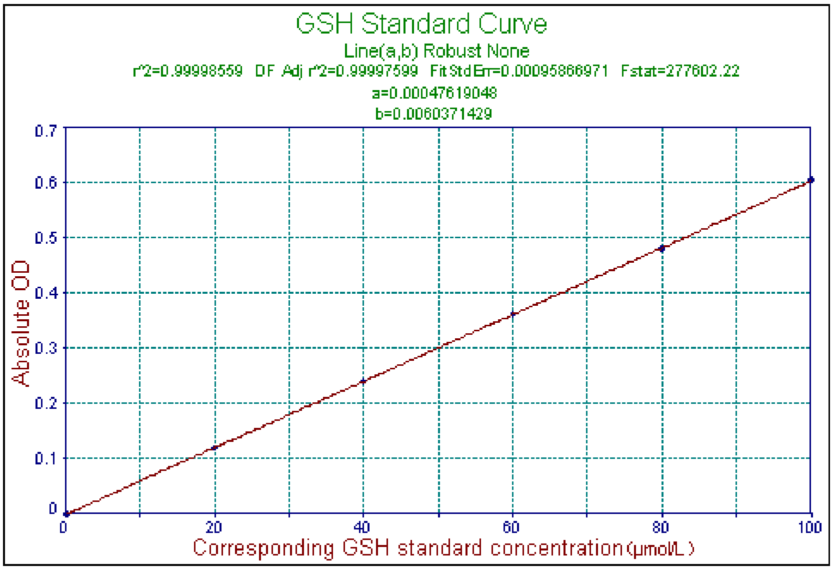

Address:Central Road, Xuanwu District, Nanjing Xinliji House1758-27 Postal code:210009 Fax:025-83227943
E-Mail:njjcbio@vip.163.com Tel:025-83360321、83360969、83551389 Technology:025-83360272
