# Supplementary material for: Dietary Klebsormidium sp. Supplementation Improves Growth Performance, Antioxidant and Anti-Inflammatory Status, Metabolism, and Mid-Intestine Morphology of Litopenaeus Vannamei
Source: Front Nutr. 2022 May 12;9:857351. doi: 10.3389/fnut.2022.857351 (PMC9136981; doi:10.3389/fnut.2022.857351)
Supplement: Supplementary file 4 [file Data_Sheet_4.pdf]

Total antioxidant capacity(T-AOC) Assay Kit

（A015-2-1 ABTS method 100T ）

1、Reagents composition & Preparation

<table>
<tr><th>Reagents</th><th>Composition</th><th>Position</th><th>Storage</th></tr>
<tr><td>R1</td><td>Check buffer</td><td>20mL×1 bottle</td><td>Store at -20℃</td></tr>
<tr><td>R2</td><td>ABTS solution</td><td>1mL×1 vial</td><td>Store at -20℃
and preserve avoid the light</td></tr>
<tr><td rowspan="2">R3</td><td>Substrate solution</td><td>0.5mL×1 vial</td><td>Store at -20℃</td></tr>
<tr><td colspan="3">Preparation of reagent 3 application solution: Dilute the solution with distilled water (40 times) at the ratio of 1:39 before use, and prepare it now</td></tr>
<tr><td colspan="4">ABTS working solution: According to R1: R2: R3 application liquid 76:5:4 proportion configuration ABTS working liquid, prepare how much you need to use, store at room temperature and preservation avoid light ,use within 30 minutes.</td></tr>
<tr><td rowspan="2">R4</td><td>Enzyme solution</td><td>0.2mL×1 vial</td><td>Store at -20℃</td></tr>
<tr><td colspan="3">Preparation of reagent 4 application solution: According to the ratio of enzyme solution: R1=1:9,the Reagent 4 application solution is prepared now, prepare when you need to use</td></tr>
<tr><td>R5</td><td>10mM Trolox solution</td><td>0.1mL×1 vial</td><td>Store at -20℃
and preserve avoid the light</td></tr>
<tr><td colspan="4">It comes with a disposable 96-hole plate</td></tr>
</table>

2、Intended use

This kit is used to measure the total antioxidant capacity of serum, plasma, tissue homogenate, cells (or supernatant) and so on.

3、Assay principle

ABTS will oxidize to green $ABTS^{\cdot +}$ under the action of the appropriate oxidant. In the presence of antioxidants, the production of $ABTS^{\cdot +}$ is inhibited. The total antioxidant capacity can be determined by measuring the absorbance at 405nm or 734nm.Trolox is a VE analogue that has similar antioxidant activity to VE, so it is commonly used as a reference for the total antioxidant activity of other

antioxidants. For example, the total antioxidant capacity of Trolox is 1. At the same concentration, the antioxidant capacity of other substances is expressed as a multiple of the antioxidant capacity of Trolox compared to Trolox.

$$ABTS^{\cdot+} \xrightarrow{Antioxidant} ABTS$$

4、Applicable equipment

Various types of photometer or microsamples can be determined.

5、Sample preparation

1、Preparation of Serum, Saliva, urine, supernatant and other liquid samples：

Blood samples should be separated to get serum or plasma in time after collection. hemolysis should be avoided during sampling. Direct determination of saliva, urine and supernatant of cells. **Plasma is recommended for Heparin or Sodium Citrate anticoagulation and should not be treated with EDTA.**

2、Tissue：

The weight of the tissue was measured accurately, and the ratio of weight (g) : Volume (ml) 1:9, adding 9 times volume of normal saline, mechanical homogenization under ice-water bath condition, fully crushing the cells to release the antioxidants, 12,000 rpm, centrifugation for 5 minutes, and take the supernatant to determination.

3、Cell:

The collection of not less than 1 million cells is recommended for cell scraping and should not be digested with trypsin and EDTA. Add 200μLcold PBS, homogenate or ultrasound to fully crush the cells and release their antioxidants, centrifuged at 4℃,12000rpm for 5 minutes, and take the supernatant to determination.

Note: The protein concentration of tissue or cell samples after homogenization can be measured by our A045-2 coomassie brilliant blue protein quantitative test kit or A045-3 BCA protein quantitative test kit.

6、Operation Procedure

	Blank	Standard	Sample
Distilled water（μL）	10		
Different concentrations of MTrolox solution（μL）		10	
Sample（μL）			10
R4 application solution（μL）	20	20	20
ABTS working solution（μL）	170	170	170
The OD values of each hole were determined by enzyme-labeled method at 405 nm (or within 405-425 nm) after 6min reaction at room temperature			

Note： After the reaction as soon as possible reading, standard Trolox solution with distilled water diluted to 0.1,0.2,0.4,0.8,1.0 mm concentration of the standard curve. The Standard Curve is done only once (see appendix I)

7、Calculate：

Take the standard OD value as the horizontal coordinate and the standard OD concentration as the vertical coordinate to make the Standard Curve. Get the curve formula by drawing software (or excel table),Take the OD value measured by the sample into the calculation formula , and then obtain the results.

8、Reference Range：

The T-AOC of human serum was 0.5～2mM;The T-AOC of human urine was 0.2-3mM

（This reference value is for reference only. It is suggested that each laboratory should establish its own reference value range）

9、Expression of T-AOC：

When Trolox is used as a standard for the determination of total antioxidant Capacity, the antioxidant Capacity of the sample can be expressed by Trolox-Equivalent Antioxidant Capacity (TEAC) .

For liquid samples (such as serum, Saliva, urine, etc.), the total antioxidant capacity can be directly expressed as the molar concentration of Trolox with the same inhibition rate, and for some samples that require dilution (total antioxidant capacity is greater than 1mM) , the dilution is multiplied

by its dilution multiple.

For a cell or plant tissue sample, if the homogenate has the same inhibitory rate of Trolox as a molar concentration, then the molar concentration of Trolox divided by the protein concentration of the homogenate is used in the calculation, the final result was expressed in mmol/g protein.

For some extracts (soluble) samples, if the extracted solution has the same inhibition rate to Trolox at a molar concentration, then the molar concentration of Trolox at that concentration is calculated by dividing by the mass concentration of the extract, the final result was expressed in mmol/g.

10、Calculation example：

Take a sample of human serum (random)10 l of the original solution, according to the above-mentioned Operation Table, and determinate the standard curve at the same time, the OD value of human serum was 0.3866,replace the OD value with the standard curve and the result is as follows:

$$\begin{aligned}\text{The T-AOC of human serum (m}M\text{)} &= -1.1214 \times \text{Assay } OD + 1.1262 \\ &= -1.1214 \times 0.3866 + 1.1262 = 0.6926\text{mM}\end{aligned}$$

Therefore, the T-AOC of this person's serum sample is equivalent to 0.6926 mM Trolox

11、Points for attention：

1、If the sample has high fat (or turbidity) , hemolysis (or special color) and other interference, each sample should be made a self-control hole (i. e. 10 l sample + 180 l distilled water or PBS mixed, leave for 6 Min, read the absorbency at 405 nm) , when calculating, the absorbance value of each sample measuring hole should be subtracted from the absorbance value of each sample corresponding to its own control hole, and then the absorbance value of each sample measuring hole should be calculated by the standard curve.

2、The concentration of Reagent 3 will decrease when the solution is exposed to light or stored for a long time, which will lead to a linear decrease of the standard curve, before the test, the result can be judged by the absorbance value of 3 points (0.4 mm, 0.8 mm, 1 mm) after the test curve. (If the absorbance value does not change or is not linear when the standard concentration increases, the dilution multiple of the three application solutions must be reduced before the test.). Reagent tridilution can generally be adjusted to 30 or 25 times.

Appendix I：Making of standard curve

1、Sample preparation:

Take The 10 mM Trolox standard solution, and dilute it with distilled water to 0.1 mM, 0.2 mM, 0.4 mM, 0.8 mM and 1.0 mM for making standard curve.

2、Operation table：

	Blank	Standard
Distilled water（μL）	10	
Different concentrations of MTrolox solution（μL）		10
R4 application solution（μL）	20	20
ABTS working solution（μL）	170	170
The OD values of each hole were determined by enzyme-labeled method at 405 nm (or within 405-425 nm) after 6min reaction at room temperature		

3、Results：

**Standard concentration (mM)**	**OD values**
0	1.0324
0.1	0.9098
0.2	0.8132
0.4	0.6272
0.8	0.2706
1.0	0.1431

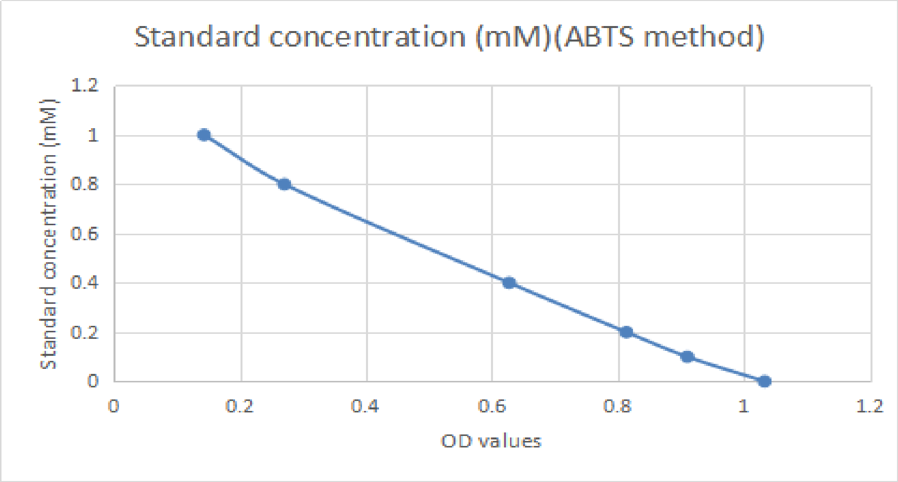

Address:Central Road, Xuanwu District, Nanjing Xinliji House 258-27 Postal code:210009 Fax:025-83227943
E-Mail:njjcbio@vip.163.com Tel:025-83360321、83360969、83551389 Technology:025-83360272
